# Transcriptome Responses of Wild *Arachis* to UV-C Exposure Reveal Genes Involved in General Plant Defense and Priming

**DOI:** 10.3390/plants11030408

**Published:** 2022-02-02

**Authors:** Andressa Cunha Quintana Martins, Ana Paula Zotta Mota, Paula Andrea Sampaio Vasconcelos Carvalho, Mario Alfredo Saraiva Passos, Marcos Aparecido Gimenes, Patricia Messenberg Guimaraes, Ana Cristina Miranda Brasileiro

**Affiliations:** 1 Embrapa Genetic Resources and Biotechnology, Brasília 70770-917, DF, Brazil; andressa.cqm@gmail.com (A.C.Q.M.); anazottamota@gmail.com (A.P.Z.M.); paulaasvc@gmail.com (P.A.S.V.C.); mario.saraiva@embrapa.br (M.A.S.P.); marcos.gimenes@embrapa.br (M.A.G.); patricia.guimaraes@embrapa.br (P.M.G.); 2 National Institute of Science and Technology—INCT PlantStress Biotech—EMBRAPA, Brasília 70770-917, DF, Brazil; 3 CIRAD, UMR AGAP, F-34398 Montpellier, France; 4 Instituto de Biociências, Department de Genética, Universidade Estadual Paulista (UNESP), Botucatu 70770-917, SP, Brazil

**Keywords:** ultraviolet light, meta-analysis, abiotic stress, biotic stress, drought, *Meloidogyne arenaria*, *Cercosporidium personatum*

## Abstract

Stress priming is an important strategy for enhancing plant defense capacity to deal with environmental challenges and involves reprogrammed transcriptional responses. Although ultraviolet (UV) light exposure is a widely adopted approach to elicit stress memory and tolerance in plants, the molecular mechanisms underlying UV-mediated plant priming tolerance are not fully understood. Here, we investigated the changes in the global transcriptome profile of wild *Arachis stenosperma* leaves in response to UV-C exposure. A total of 5751 differentially expressed genes (DEGs) were identified, with the majority associated with cell signaling, protein dynamics, hormonal and transcriptional regulation, and secondary metabolic pathways. The expression profiles of DEGs known as indicators of priming state, such as transcription factors, transcriptional regulators and protein kinases, were further characterized. A meta-analysis, followed by qRT-PCR validation, identified 18 metaDEGs as being commonly regulated in response to UV and other primary stresses. These genes are involved in secondary metabolism, basal immunity, cell wall structure and integrity, and may constitute important players in the general defense processes and establishment of a priming state in *A. stenosperma*. Our findings contribute to a better understanding of transcriptional dynamics involved in wild *Arachis* adaptation to stressful conditions of their natural habitats.

## 1. Introduction

During evolution, land plants developed mechanisms to cope with various and unpredictable abiotic and biotic stresses to which they are continuously exposed under habitat-imposed growth conditions. As part of the inducible stress defense mechanisms, plants can remember experienced stress events and prepare (prime) faster and stronger responses to react more efficiently to the next upcoming stress challenge. This stress memory process is known as “priming” and has been recently explored to develop stress-resistant crops with improved ability to adapt to, or withstand, suboptimal growth conditions under multiple stresses [1]. The mechanisms behind stress memory acquisition are largely unclear but have been associated with a series of physiological, biochemical, and molecular processes such as epigenetic regulation, chromatin pattern, transcriptional initiation, primed conformation of proteins, and metabolic or hormonal signatures [2].

Recent studies showed that “memory genes”, a subset of genes highly responsive to a primary stress event, maintained altered expression patterns in subsequent stress events and across generations [3,4,5,6]. Evidence has even suggested that the reprogrammed transcriptional responses are one of the most likely aspects that leads to the acquisition of stress memory in plants [7,8]. Memory genes are related to general defense response processes, including reactive oxygen species (ROS) homeostasis, flavonoid, proline and sugar metabolism, induction of systemic signaling by RbohD, activation of pathogen-related proteins and hormonal signaling pathways [9,10,11]. These non-specific defense processes are known to connect broad-spectrum responses and crosstalk between abiotic and biotic stresses, bringing plants to a primed state, in which they respond more effectively to subsequent stress exposure. Nonetheless, in primed plants, the costly energy investments and trade-offs associated with the need for a rapid defense response are not imposed without compromising much on productivity [12].

The priming effect of ultraviolet (UV) radiation of seeds has long been adopted by farmers as an effective and low-cost technique (named on-farm seed priming) to improve germination rate, seedling vigor, stress tolerance at later developmental stages, and, ultimately, better crop yields [13]. In addition to seeds, the exposure of fruits, flowers, tubers, and vegetables to low doses of UV is also a very efficient strategy used by producers in delaying senescence, improving nutritional content, and inhibiting the development of pre- and post-harvest diseases that reduce losses and waste of fresh produce [14]. These adaptive responses induced by UV treatment occur in the framework of the biological phenomenon known as UV hormesis and trigger both stress and antioxidative signaling in plants, with enhanced generation of ROS and production of secondary metabolites and synthesis of non-enzymatic and enzymatic antioxidants. Consequently, plants develop improved tolerance to environmental constraints and resistance against pest and pathogen attacks by stimulating the local or systemic expression of stress memory genes [15,16,17]. Recently, Agathokleous and co-workers [18] postulated that plant priming is preconditioning, which itself is a manifestation of hormesis through the activation of stress adaptive responses.

Although one of the most adopted strategies for stress-induced priming in plants, few studies have addressed the reprogrammed transcriptional responses after UV-C exposure from a transcriptomic point of view [8,19,20]. Recently, our group demonstrated that wild *Arachis* plants exposed to UV-C produce high levels of resveratrol, which is associated with the induced expression of resveratrol synthase genes [21]. Cultivated peanut (*Arachis hypogaea*) and its wild relatives are among the few plants that naturally synthesize resveratrol, an important phytoalexin selectively accumulated in response to pathogen attack [22]. It is well-established that enhanced accumulation of resveratrol, and other phenolic compounds, plays a direct role in defense responses against a wide range of plant pathogens and can be considered as markers for plant disease resistance [23]. Besides being a high resveratrol producer, wild *A. stenosperma* is highly resistant to a diversity of pathogens, including the root-knot nematode (RKN) *Meloidogyne arenaria* and fungi causing foliar diseases (*Puccinia arachidis* and *Cercosporidium personatum*) [24,25,26], and also tolerant to many environmental constraints [27,28,29].

The present study investigated the global transcriptomic changes induced by UV exposure in detached leaves of *A. stenosperma* to understand better the molecular mechanisms involved in activating plant defense responses to primary stress events and the potential acquisition of a priming state. Detached leaves have long been successfully used as explants for transcriptomic, proteomic, and metabolomic analysis of stress responses induced by UV exposure in leaves of cultivated and wild *Arachis* species [21,22,30,31], as well as in several plant species, including grapevine [32,33], soybean [34], *Polygonum cuspidatum* [35], *Arabidopsis thaliana* [36], spinach [37], rice [38], and maize [39]. The controlled environment of detached-leaf assays can overcome limitations imposed by field and greenhouse evaluations conducted with whole plants, allowing more standardized assessments and improving the number and homogeneity of samples that could be evaluated. Detached-leaf assays are particularly a practical and suitable experimental procedure for a heterogeneous group of plants, which is intrinsically the case for several wild, perennial, and/or large-size species, showing significant data correlations with whole-plant assays [40,41,42,43].

The focus of our work was placed on *A. stenosperma* as wild species model that harbors resistance/tolerance to different types of biotic and abiotic stresses. Global transcriptome analysis revealed changes in stress-related pathways involved in broad-spectrum defense responses and identified putative stress memory genes induced by UV stimulus and other primary stresses. Thus, our study allowed a comprehensive understanding of UV-induced defenses in wild *Arachis* that could provide insights into the genetic basis of wild species adaptation to their natural habitats and help elucidate the involvement of priming memory in their survival in stressful environments.

## 2. Results

### 2.1. RNA-Seq Data Analysis

Illumina HiSeq 2500 sequencing of cDNA libraries from *A. stenosperma* non-treated (CTR) and UV-treated (UV) samples generated over 36 and 38 million raw reads, respectively (Table S1). The *A. stenosperma* raw reads were then successfully quantified into the reference genome of *Arachis duranensis* (Available online: http://peanutbase.org/ (accessed on 11 September 2021)) where they illuminated 24,113 and 24,978 gene models in CTR and UV libraries, respectively (Table S1). From that, a total of 27,233 unique gene models were generated, representing 74.1% of the 36,734 protein-coding genes predicted for *A. duranensis* [44].

**Figure 1:**
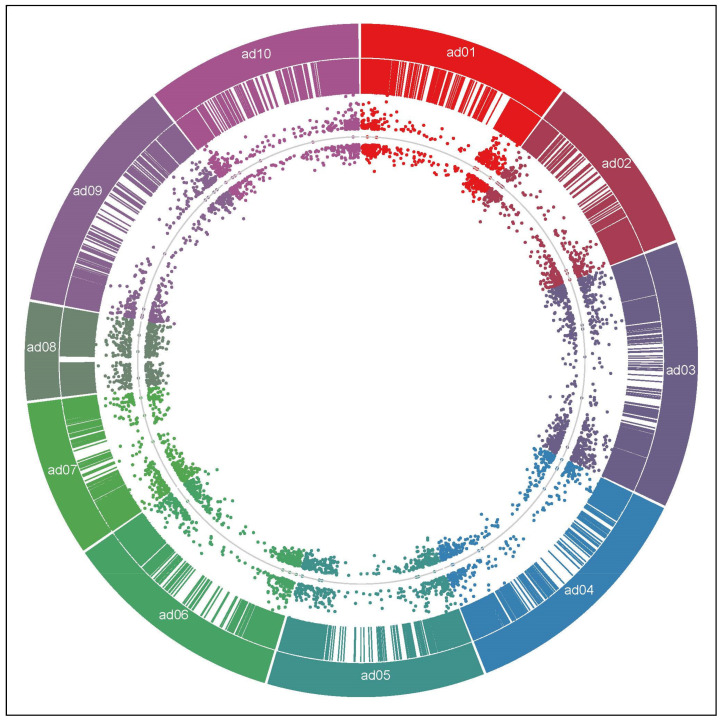
Distribution of DEGs on the *Arachis duranensis* chromosomes. Distribution of the 5751 *A. stenosperma* DEGs (colored lines) in the ten chromosomes of *A. duranensis* (ad01 to ad10). The inner dots represent the distribution of log2FC values for each up- and downregulated DEG, with the line indicating log2FC = 0.

Comparison between CTR and UV libraries sequence data was used to infer in silico differential expression, and 5751 Differentially Expressed Genes (DEGs) were assigned as showing statistical significance at adjusted *p*-value (FDR) < 0.05 and expression fold change (FC) between UV and CTR samples of at least four [log2FC (UV/CTR) > 2 or <−2] (Table S1). A similar distribution between upregulated (2679; 46.6%) and downregulated (3072; 53.4%) *A. stenosperma* DEGs was observed in response to UV-C exposure (Figure 1; Table S1). The 5751 DEGs were unevenly distributed in the ten chromosomes of the *A. duranensis* reference genome, being more frequently placed on distal chromosomal regions (Figure 1). The high density of protein-coding genes at the distal ends of chromosomes agrees with the gene-rich characteristic of these recombination hotspot regions in wild *Arachis* genomes [44]. It was observed an overall high amplitude of differential expression of the 5751 DEGs (Figure 1), as corroborated by the great number of 3215 DEGs with at least 8-FC (log2FC > 3 or <−3) and 1809 with 16-FC (log2FC > 4 or <−4), being similarly up- and downregulated (Table S1).

### 2.2. Functional Enrichment Analysis of DEGs

**Figure 2:**
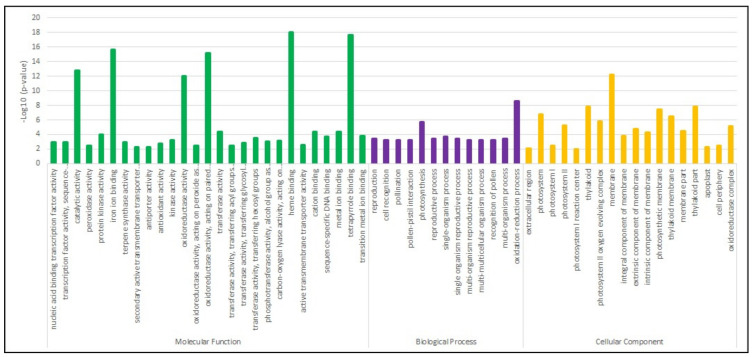
Gene Ontology (GO) enrichment of *Arachis stenosperma* DEGs in response to UV-C exposition.

Gene Ontology (GO) terms enrichment analysis was performed for the 5751 DEGs that could be assigned to the three main GO functional categories, being the molecular function the category with more enriched terms (27), followed by cellular component (18) and biological process (13) (Figure 2). The molecular function category showed the terms with the highest *p*-values (>12) of -log10, such as catalytic activity, iron ion binding, oxidoreductase activity, heme and tetrapyrrole binding, which could be involved in triggering the oxidative and stress responses. In addition, terms associated with transcription factor and kinase activities, both associated with defense priming, were also enriched (Figure 2). In the biological process category, two terms (photosynthesis and oxidation-reduction process) showed *p*-values higher than 5.6, whereas the other terms were similarly enriched with *p*-values around 3 (Figure 2). Both molecular function and biological process categories agreed with the most enriched cellular component terms found in this analysis (photosystem; membrane- and thylakoid-associated terms), which are also important components in general plant responses towards stresses (Figure 2).

### 2.3. Functional Characterization by Mapman Analysis

**Figure 3:**
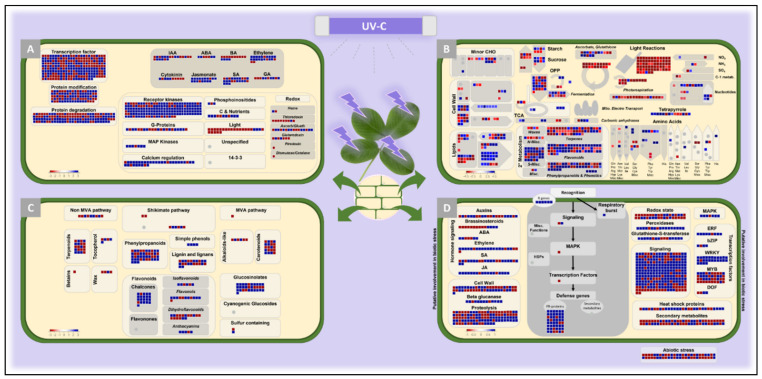
MapMan functional classification analysis of *Arachis stenosperma* DEGs. Functional overview of *A. stenosperma* DEGs related to the plant cell regulatory (**A**), general metabolic (**B**), secondary metabolic (**C**), and biotic stress-related (**D**) pathways. Blue and red dots mean up- and downregulated DEGs, respectively, in response to UV-C treatment.

To get an insight into the functions of the *A. stenosperma* genes responsive to UV, we conducted a MapMan analysis based on the functional ontology of DEGs. In total, 3120 unique DEGs (54%) could be assigned to at least one MapMan functional category. The MapMan analysis of main plant cell regulatory pathways (Figure 3A) indicated that most unique DEGs (892; 28.6%) are involved in cell signaling, protein dynamics, hormonal and transcriptional regulation. Genes associated with receptor kinases and calcium regulation as well as JA, SA, and ethylene signaling are particularly induced in response to UV, whereas light-related genes are mostly downregulated (Figure 3A). Accordingly, the global view of DEGs involved in diverse metabolic pathways showed that photosynthesis was clearly inhibited by UV treatment, whereas genes associated with secondary metabolism were enriched mostly in the terpenoid, phenylpropanoid, glucosinolate, and flavonoid pathways (Figure 3B,C). Likewise, transcripts related to “cell wall” (47) and “lipids” (80) categories were also differentially regulated under this condition (Figure 3B).

**Figure 4:**
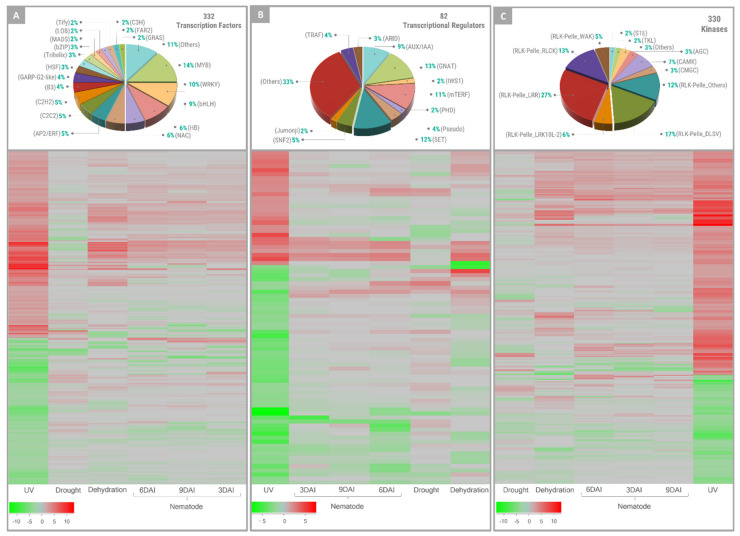
Distribution of *Arachis stenosperma* DEGs into plant regulator gene families. Distribution of *A. stenosperma* DEGs into gene families of transcription factors (**A**), transcriptional regulators (**B**), and protein kinases (**C**) during the response to UV-C exposure and heatmap of their relative expression under six stress treatments, with normalized log2FC values in a red-green scale.

A more detailed analysis of highly enriched terms in the MapMan categories, such as transcription factors (TFs), transcriptional regulators (TRs), and protein kinases (PKs), was also conducted, using specific *Arachis* transcriptome databases. These functional categories are largely involved in the transcriptional reprogramming of the enhanced primed phase of plants upon stress and could be considered common priming state indicators [8]. As expected, many TFs (332) showed an altered expression in response to UV stress, representing 18% over the 1854 gene models coding for TFs in *A. duranensis*. These UV-responsive TFs belong to 38 families, being MYB (14%), WRKY (10%), and bHLH (9%) the most represented (Figure 4A). Furthermore, most of the 332 TF-encoding DEGs (56%) are upregulated in response to UV, particularly those belonging to the WRKY family that had 31 out of 32 members activated, representing 36% of the 87 WRKY-encoding genes identified in *A. duranensis*. This marked activation of WRKY genes suggests that this TF family, unique to plants, plays a major role in the transcriptional reprogramming in response to UV. Accordingly, WRKY was also identified as one of the most abundant TFs that exhibited modulations in transcript levels as part of the response of wild *Arachis* to abiotic and biotic stresses [28,45,46]. In contrast, DEGs belonging to B3 and HB TF families were mainly downregulated in response to UV (78% and 67%, respectively).

For plant regulators (Figure 4B), a total of 82 DEGs belonging to 16 TR families were identified, mainly being (54; 66%) downregulated. In particular, the expression of all nine mTERF family members was negatively regulated by UV stress, as well as the majority GNAT and SET family representatives (91% and 80%, respectively) (Figure 4B). The remaining classes were equally composed of both up- and downregulated TRs.

The enhanced activation of kinases is part of the transcriptomic changes that mark the priming phase in different plant species [8]. UV exposure also altered the expression levels of a high number of PKs (330), which represent 28% of all 1156 kinases described for *A. duranensis*, with the majority (222; 67%) being activated (Figure 4C). Almost 80% of these 330 PK-encoding DEGs belonged to the receptor-like kinase (RLK)/Pelle gene family, the largest class of PKs (Figure 4C), and predominantly (185; 71%) upregulated in response to UV. This group is stress-specific defense-related PKs that facilitate a signaling cascade for plant adaptation under various stresses [47]. The Mitogen-activated protein kinases (MAPKs), another family of PKs that play diverse roles in response to abiotic and biotic stresses, had three members activated by UV exposure and only one downregulated.

The in silico expression profiles of the TFs, TRs, and PKs *A. stenosperma* DEGs in response to UV were also analyzed and compared to those previously obtained by our group in response to other five biotic and abiotic stress treatments [28,46,48] (Figure 4). In general, the expression profiling of *A. stenosperma* regulator genes across these different stress treatments revealed that they were strongly affected by UV exposure since the magnitude of expression change was far greater than that observed for the other stresses (Figure 4).

### 2.4. NBS-LRR Genes Responsive to UV Exposure

**Figure 5:**
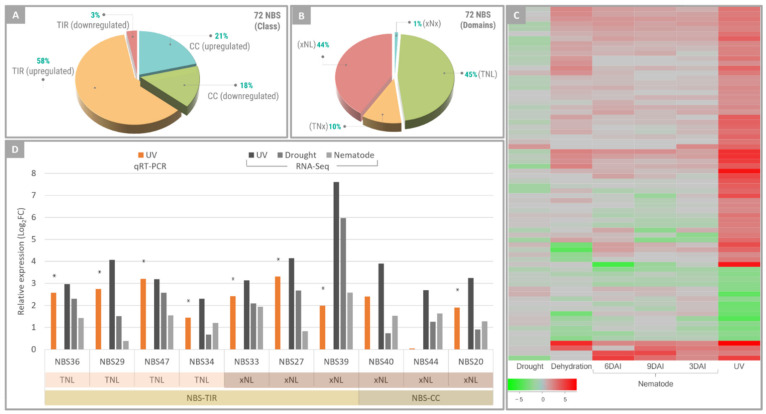
Distribution of *Arachis stenosperma* DEGs into NBS-LRR sub-families. Distribution of *A. stenosperma* NBS-LRR sub-families expressed in response to UV-C exposure (**A**,**B**). Heatmap of the relative expression of the 72 *A. stenosperma* NBS-LRR genes under six stress treatments, with normalized log2FC values in a red-green scale (**C**). Expression profile (log2FC) of ten *A. stenosperma* NBS-LRR genes analyzed by qRT-PCR and RNA-Seq (**D**). Asterisks mean significantly regulated genes.

MapMan analysis of DEGs also revealed that biotic stress-related pathways were significantly affected in *A. stenosperma* leaves following UV stimulus (Figure 3D). In particular, a relatively high number (72) of NBS-LRR (nucleotide-binding site and leucine-rich repeats) genes was identified as DEGs, which represents 21% of the total (345) NBS-LRR gene models described for *A. duranensis* [44]. Furthermore, most of these DEGs encoding NBS-LRRs (79%) were upregulated in response to UV (Figure 5). This result is in accordance with our previous studies, showing that 15% and 10% of the NBS-LRRs identified in *A. stenosperma* transcriptome surveys were regulated upon infection with *M. arenaria* and *C. personatum*, respectively [45,48].

The majority (61%) of NBS-LRRs responsive to UV belonged to the TIR-type (Figure 5A). Their further classification into subfamilies, based on the presence of three conserved domains alone or in combination [49], showed that they were similarly distributed between TNL and xNL subfamilies (45 and 44%; respectively), while only seven genes were identified in TNx subfamily and a single one in xNx (Figure 5B). The two most expressed NBS-LRR subfamilies in response to UV exposure were those previously shown to be most responsive to RKN infection, with TNL corresponding to 30% and xNL 55% of the DEGs [48].

A comparative expression analysis of the 72 *A. stenosperma* DEGs encoding NBS-LRRs in response to UV showed that all these genes were also responsive to other biotic and abiotic stresses previously studied by our group (Figure 5C). Albeit not always constituting DEGs in previous transcriptome analyses, the great majority of these genes was upregulated under all stresses (Figure 5C), with the magnitude of the response to UV exposure being consistently higher than to the other stress treatments, regardless of their behavior (up- or downregulation) (Figure 5C). As previously observed for soybean (*Glycine max*) and grapevine (*Vitis vinifera*) [50,51], UV radiation can have substantial effects on the expression of some NBS-LRR encoding genes. Here, we found that in *A. stenosperma*, the UV-responsive NBS-LRRs were also modulated by other biotic and abiotic stresses, although displaying lower expression levels (Figure 5C).

To further analyze these effects, a more detailed expression analysis by qRT-PCR was carried out using a subset of ten NBS-LRR genes (Table S2), comprising different subfamilies (TNL and xNL) and showing distinctly in silico expression magnitudes. As a whole, the expression behavior of all of the ten NBS-LRR representatives in response to UV inferred from in silico analysis was validated by qRT-PCR (Figure 5D), with all genes displaying upregulation behavior, with variable expression levels depending on the stress imposed (UV, drought or nematode infection) (Figure 5D). These findings suggest that some NBS-LRR genes, which are a class of R genes generally triggered by specific biotic stresses in *A. stenosperma* [45,46,48,52], can also respond to more severe and unspecific stresses, such as UV and dehydration, and therefore be associated to the general resistance/tolerance of this wild species to stressful conditions.

### 2.5. Comparative Transcriptome Meta-Analysis of A. stenosperma under Different Stresses

**Figure 6:**
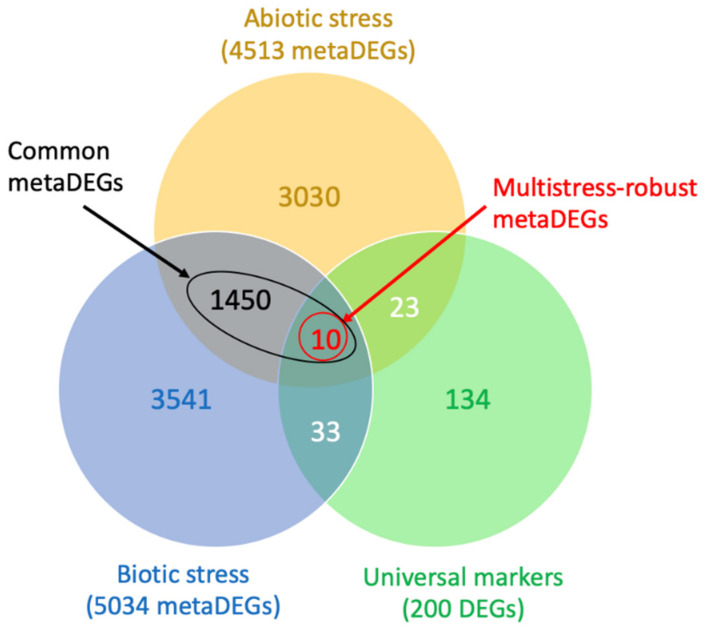
Venn-diagram of *Arachis stenosperma* DEGs in response to abiotic and biotic stress treatments. Intersections among *A. stenosperma* metaDEGs in response to biotic stress treatments (nematode infection; blue) and abiotic stress treatments (UV exposure, dehydration and dry-down; yellow); and universal gene markers of plant priming (green). Four RNA-Seq libraries from biotic and six from abiotic stress treatments were used for the comparative transcriptome meta-analysis (Table S3). A total of 1460 metaDEGs shared among all biotic and abiotic analyzed studies were considered as ‘common metaDEGs’ and ten of them were identified as ‘multistress-robust metaDEGs’.

Aiming to identify genes that are commonly responsive to a broad spectrum of primary stimulus, we performed a meta-analysis comparison of abiotic and biotic stress responses in *A. stenosperma* by analyzing our previous transcriptomic datasets (Table S3) [28,46,53]. For meta-analysis, the six RNA-Seq libraries from abiotic stress treatments (UV exposure, dry-down, and dehydration) were analyzed separately from those of the four biotic stress treatments (nematode inoculation) (Table S3). Overall, we found 4513 metaDEGs commonly regulated, showing the same expression trend (up- or downregulation) in response to abiotic stresses and 5034 upon biotic stresses (Figure 6). Of these, 1460 (18%) metaDEGs were shared among all biotic and abiotic analyzed studies. They were thus considered here as common metaDEGs responsive to a wide range of stresses in *A. stenosperma* (Figure 6), being ten of them further identified as ‘multistress-robust metaDEGs’ (see below). This result indicated that most DEGs were uniquely modulated by either abiotic or biotic stress, and *A. stenosperma* may use distinct transcriptional mechanisms to deal with different stresses, as previously reported in wild *Arachis* species plants submitted to individual and combined stresses [53]. This metaDEGs dataset was further screened for putative plant priming genes responsible for initiating non-specific defense responses in many species.

### 2.6. Identification of Gene Markers of Plant Priming

In an attempt to investigate genes involved in the *A. stenosperma* transcriptional dynamics after UV exposure and putatively associated with defense priming, we searched for orthologs of the universal gene markers of plant priming as established by Baccelli and co-workers [8]. These 44 *Arabidopsis thaliana* genes were considered reliable markers of the priming phase through meta-analysis of RNA-Seq data obtained during different priming conditions and in different plant species. For the orthologs search, we explored a set of protein orthogroups shared among 22 plant species, including *A. duranensis*, previously characterized by our team [54]. This comparative analysis allowed the identification of orthologs between the *A. thaliana* universal gene markers [8] and the *A. duranensis* gene models significantly differentially expressed (FDR < 0.05) in response to UV (Table S1). Overall, we found 37 out 44 *A. thaliana* gene markers that shared orthogroups with at least one *A. duranensis* gene model (Table S4). This result suggests that UV-C stimulus stress triggered in *Arachis* changes in the expression of 84% of the genes considered universal markers of the priming phase. Moreover, most of the 200 genes belonging to the above priming-markers orthogroups displayed the same trend (up- or downregulation) as the other four plant species (banana, tomato, grapevine, and *A. thaliana*) primed with four different stimuli, according to [8]. Interestingly, two of the three largest orthogroups (OG0000001 and OG0000057) encoding both for protein kinases (Table S4) were also previously identified as orthogroups commonly regulated upon nematode infection in four RKN-resistant plant genotypes (*A. stenosperma,* coffee, soybean, and rice) [54]. Furthermore, we identified ten metaDEGs belonging to six orthogroups shared with *A. thaliana* universal gene markers (Figure 6; Table 1), suggesting that they are regulated at the priming phase in *A. stenosperma.* These ten metaDEGs are associated with general stress-defense responses in plants, such as receptor kinases, MYB-like TF, and epoxide hydrolase (Table 1). Based on previous study [55], we considered these ten genes as ‘multistress-robust metaDEGs’ (Figure 6).

### 2.7. qRT-PCR Expression Analysis

More detailed expression analysis was conducted by qRT-PCR for another 18 *A. stenosperma* previously associated with priming phase (Table S2), such as genes involved in secondary metabolism (IRL, STS, UGT, BBE, N8DT, and PAL); cell wall structure and integrity (EXLB, EGC, FLA, LAC, and PRP); basal defense pathways (eGCL, NUDT, SYP); or stress signal transduction (LOB, AKT) [8,19,55,56,57,58,59,60]. These candidate genes were selected among the 1460 metaDEGs identified here as commonly regulated by a broad range of primary stresses (Figure 6). In addition to UV exposure, the expression behavior of these 18 candidate genes was also analyzed in *A. stenosperma* RNA samples previously collected from roots subjected to dry-down treatment (drought) [61] or infection with *M. arenaria* [62] and leaves challenged with *C. personatum* [45].

**Figure 7:**
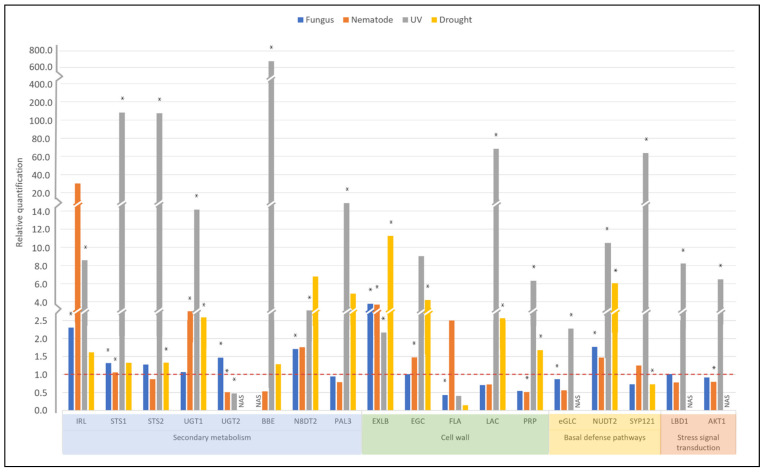
Expression of *Arachis stenosperma* DEGs as determined by qRT-PCR. Expression profiles of 18 DEGs identified in *A. stenosperma* plants in response to four stress treatments: inoculation with *Cercosporidium personatum* (fungus; blue) and *Meloidogyne arenaria* (nematode; orange), UV-C exposure (UV; gray), and dry-down imposition (drought; yellow). The relative quantification (RQ) of mRNA levels of DEGs in stressed samples was normalized with non-stressed control samples using two *Arachis* reference genes (60S and GAPDH), with RQ values above or below 1.0 indicating, respectively, up- or downregulated DEGs (dashed red line). Asterisks mean significantly regulated genes.

All selected metaDEGs analyzed by qRT-PCR showed specific transcript amplification with high amplification efficiencies. The qRT-PCR expression patterns of these genes were highly related to their in silico analysis, with the majority being positively regulated in response to most stresses (Figure 7). Overall, genes involved in secondary metabolism were highly induced in *A. stenosperma* by the four stresses, particularly in response to UV exposure (IRL, STS1 and 2, UGT1, BBE, N8DT2, and PAL3). The exception was UGT2 that was downregulated upon UV and nematode stresses. BBE gene, encoding for the Berberine Bridge enzyme-like, showed a strong upregulation of 740-fold in response to UV, which was the greatest change in expression among all analyzed genes and stresses (Figure 7). A remarkable transcriptional induction by UV (>139-fold) was also observed for both STS genes. Genes encoding for cell wall modifying proteins (EXLB and EGC) were upregulated by both biotic and abiotic stresses, while genes involved in the cell wall structure and formation (LAC and PRP) were induced by UV and drought but downregulated in response to both pathogens (nematode and fungus). Likewise, the expression of the FLA gene decreased in response to three stresses (fungus, UV, and drought) and increased only upon nematode infection (Figure 7). UV and pathogen stresses seemed important for regulating UGT2, eGLC, LBD1, and AKT1 gene expression, as they were not detected in response to drought. The SYP121 gene was highly upregulated under UV stress (>64-fold), while the NUDT2 gene was induced to a lesser extent in all stresses studied (Figure 7).

## 3. Discussion

The genus *Arachis* is native to South America, where wild species evolved adaptive traits to survive through time under multivariate ecological conditions in their native habitats, comprising savannah-like regions, partially flooded areas, and subtropical forests, in altitudes from sea level to the Andes Mountains [63]. In particular, *A. stenosperma* seems to have been dispersed and selected by native indigenous given its relatively good yield [27] and shows high levels of tolerance/resistance against pests and environmental hazards [25]. Inevitably, most of the potentially useful stress-resilience traits harbored by wild relatives were lost during domestication and are no longer available in the currently cultivated peanut (*A. hypogaea*) primary gene pool [64].

Over the past years, our research group has explored the genetic diversity from wild *Arachis* germplasm as a valuable resource for discovering useful genes and alleles to build climate-resilient crops. Through functional genomic approaches, these studies disclosed a number of *A. stenosperma* genes implicated in its natural resilience to biotic and abiotic stresses [65]. The present study is part of this effort by analyzing the transcriptional changes in *A. stenosperma* leaves upon UV-C treatment. Considering that UV radiation is a well-known elicitor of defense responses leading plants to a primed state, our findings enabled the discovery of genes potentially involved with broad-spectrum defense molecular mechanisms underlying priming in this wild species.

Several studies have elucidated general gene expression patterns behind defense priming in plants [8,19,20]. However, only recently, the comprehensive transcriptome reprogramming involved in priming by UV-C (200 to 280 nm), the shorter-wavelength portions of UV light, has been addressed. Despite the damaging and even lethal effects of these highly energetic wavelengths, there is rising evidence that UV-C exposition stimulates adaptive mechanisms in plants to deal with abiotic and biotic stresses, depending on doses and timing [15,66]. Additionally, repeated UV-B radiation treatment during few days caused a similar effect on transgenerational stress memory to the single dose of UV-C, which partially mimics the response to biotic stress [67].

Exposition to UV-C of natural resveratrol-producing plants, such as *Arachis* spp., grapevine, and *Polygonum cuspidatum*, triggers defense and general stress responses and promotes the expression of genes involved in the biosynthesis of secondary metabolites, in particular stilbene synthase (STS) genes [21,35,50]. Recently, Xu and co-workers [17] suggested that a series of transcriptional events occurred in strawberry (*Fragariae* × *ananassa*) plants primed by UV-C, mounting a synergistic response with pathogen elicitors to establish an effective resistance. There are also a series of transcriptome surveys reporting UV-C-regulated genes that could mediate induction of pathogen resistance and delay in ripening and senescence by postharvest treatments in fresh fruits and vegetables [14,15,68,69,70,71]. These genes are mainly involved with oxidative signaling, defense responses, cell wall disassembly, lipid, and sugar metabolism. Besides mimicking the pathogen-induced responses, controlled UV-C radiation is also a stressor that induces abiotic stress signaling and has the potential to stimulate plant defense to environmental constraints [13]. Indeed, seeds of lettuce (*Lactuca sativa*) and bean (*Phaseolus vulgaris*) subjected to UV-C priming treatments exhibit higher tolerance to salt stress [72,73].

In the present study, we subjected *A. stenosperma* detached leaves to low UV-C doses as a primary stress treatment capable of inducing a primed state and leading to resistance responses against biotic and abiotic stresses. A total of 5751 DEGs were identified by transcriptome analysis, representing 15.6% of the predicted protein-coding genes in the reference genome *A. duranensis*, a closely *A. stenosperma* related wild species [44]. The overall number of induced and repressed DEGs in response to UV was quite similar, while a great amplitude on the response magnitude was observed. These results reveal that UV-C radiation drastically alters gene expression behavior, producing almost four times the number of DEGs previously identified in *A. stenosperma* by other isolated stresses, such as dehydration (1247 DEGs; [28] and nematode infection (1750 DEGs; [46], or by combined abiotic and biotic stresses (1224 DEGs; [53]. Our data also agree with previous transcriptome studies showing that a large number of plant genes were differentially regulated by UV-C radiation, as for *P. cuspidatum* in which nearly 25% of the expressed genes were affected [35]. Moreover, the number of genes identified in different plants as DEGs upon UV exposure was notably higher compared to those modulated by other stimuli, such as gibberellins treatment [74], heavy metals and ionizing radiation [75], low temperature [68], and jasmonate and salicylate treatments and herbivore infestation [76].

*A. stenosperma* detached leaves were used here as explants based on the extensive literature that successfully describes its use for molecular characterization of responses to UV stress in leaves of many plant species, including wild *Arachis* [21,22,30,31,32,33,34,35,36,37,38,39]. In addition, detached-leaves assays are an accurate methodology to produce large sample sizes, strengthening the accuracy of the statistics [40,41,42,43]. However, it is important to point out that, using this approach, some of the DEGs identified as responsive to UV might also be simultaneously responsive to wounding stress that intrinsically occurred when leaves were detached from plants. To better address this point, we found that amongst the 5751 DEGs identified in our study as responsive to UV, 743 DEGs (13%; Table S5) could also be responsive to a second intrinsic stress (wounding). These *A. stenosperma* DEGs responsive to concurrent stresses (wounding and UV) were identified by searching their corresponding orthologs among the 657 *A. thaliana* genes described by Cheong and co-workers [77] as altered by wounding at steady-state mRNA levels. Previous works indicate a crosstalk between UV and wounding signaling pathways that synergistically induce the biosynthesis and accumulation of secondary metabolites and expression of genes involved in general stress responses [78,79,80,81].

### 3.1. Exposure to UV-C Resulted in Altered Expression of Genes Encoding for TFs, Regulators, Kinases, and NBS-LRRs

Functional categories analysis of DEGs indicated that general protective responses to UV-C were activated on the *A. stenosperma* leaf transcriptome, including the well-documented TFs, TRs, and PKs. As outlined above, the response of most of these regulator genes to UV was stronger than that observed for other abiotic and biotic stresses. We found that 18% of the predicted genes encoding for TFs were responsive to UV stimulus in *A. stenosperma*, with the majority belonging to MYB and WRKY families. These two TF families play critical roles in plant defense responses and are important regulators of biotic and abiotic stress tolerance [55,82]. MYB and WRKY were also among the stress memory genes identified as highly induced upon exposure of soybean and potato (*Solanum ajanhuiri*) plants to cycles of watering and water deprivation [3,58]. Opposite to the overall activation of TFs, other transcriptional regulators, such as mTERF, GNAT, and SET, were clearly repressed in response to UV exposure. Similar expression behavior of these regulators, such as upregulation of MYB and WRKY and downregulation of GNAT and mTERF family genes, was described for rice (*Oryza sativa*) seedlings exposed to chilling stress [83]. Although some recent studies supported an emerging role for mTERFs in stress tolerance, their function in plants is still poorly understood [84]. Together with these plant regulator families, kinases also act as key integrators of different stress signaling pathways and are suggested to be one of the potential mechanisms underlying plant priming and induction of systemic acquired resistance (SAR) [20,85]. Here, UV-C caused a general activation of *A. stenosperma* RLK/Pelle kinases that constitutes one of the largest subfamilies of defense-related proteins, acting as a signaling cascade to protect plants against an ever-changing population of stress elicitors [47].

Recently, a meta-analysis study identified 197 ‘multistress-robust genes’ that responded to some primary stress and remained activated in response to different combinations of abiotic and biotic stresses [55], indicating their putative role in priming defense. As observed here, these genes included several members of WRKY and kinase families in response to a variety of stresses. Moreover, one of these ‘multistress-robust genes’ encoding for an *A. thaliana* protein kinase (AT4G21390) shared an orthogroup (OG0000001) with four of the 200 *A. stenosperma* priming marker genes (Table 1).

Here, UV-C exposure was shown to have a clear effect on the expression of *A. stenosperma* NBS-LRR genes that also exhibited dynamic expression patterns in response to other biotic and abiotic stresses. In accordance with their functionality, most of the UV-induced NBS-NLR genes showed low expression levels in response to the stresses studied. High expression of NBS-LRR genes has a considerable metabolic cost and can even cause plant death through the hypersensitive response (HR) [86]. Therefore, most NBS-LRR members have their functionality only enabled when requested and with typically low expression levels. Nonetheless, NBS-LRR genes can present different expression patterns in response to specific stresses, or as a result of TFs actions [87] and non-coding RNAs (miRNA) [88], as those elicited by UV stress.

### 3.2. A. stenosperma MetaDEGs Involved in Responses to Different Stress and Putatively Associated with Defense Priming

To explore the core *A. stenosperma* transcript dynamics under biotic and abiotic stress conditions, we conducted a meta-analysis by combining the UV RNA-Seq data produced here with our previous transcriptomic datasets [28,46,53]. Considering that UV-C exposure is an abiotic stress, we analyzed this data together with the transcriptome of two abiotic stress treatments, i.e., severe (dehydration) and moderate (dry-down) drought imposition, as previously reported by [89] for wild *Arachis* plants submitted to water-limited conditions. This analysis revealed 4513 DEGs commonly modulated by these abiotic stress treatments in *A. stenosperma*. Likewise, we found that 5034 DEGs were shared by transcriptome of *A. stenosperma* during its resistance response to *M. arenaria* infection (biotic stress). The overlap of the two sets of abiotic and biotic DEGs allowed the identification of 1460 metaDEGs that are commonly responsive to all these primary stresses. This comparative meta-analysis indicated that, regardless of the number of DEGs identified in each stress analysis and type of stress imposed, the differences between gene expression responses to biotic and abiotic stresses seemed more prevalent than their similarities, suggesting distinct transcriptomic features trigged by each stress type. These findings reinforce previous meta-analysis studies showing that most plant genes were uniquely regulated by either abiotic or biotic stress [55,82,90] and corroborated our recent evidence revealing that distinct stresses prompt different sets of genes in wild *Arachis* [53].

We also analyzed a set of marker genes, defined as universally regulated during the priming phase of plants [8], to investigate *A. stenosperma* DEGs associated with defense priming in response to UV exposure. The primary stress is responsible for the onset of memory genes that are usually related to general, nonspecific defense response processes. The altered expression of these genes at the priming phase is maintained in subsequent stresses events, leading to the acquisition of a primed state [11,91]. We identified 200 DEGs that shared the same orthogroup with 37 *A. thaliana* genes (out 44) considered as reliable markers of priming [8], indicating that nearly all universal markers (84%) are modulated when a UV stimulus is applied to *A. stenosperma* leaves. Remarkably, 10 out of these 200 *A. stenosperma* markers of priming were also identified as metaDEGs and, therefore, commonly regulated by multiple primary stresses at the priming phase in *A. stenosperma*. These metaDEGs are involved in general, unspecific molecular mechanisms of stress defense (Table 1) and may be considered ‘multistress-robust metaDEGs’, as recently defined by [55]. It makes them potential molecular markers and candidate genes for biotechnological purposes to improve tolerance in plants to concomitant and recurrent stresses.

### 3.3. Secondary Metabolism Is Affected by Multiple Stresses in A. stenosperma

We also analyzed the expression behavior of 18 candidate genes described in the literature as being associated with general plant defense strategies and, particularly, molecular changes during the priming phase, which occurs immediately after the perception of a primary stress stimulus. The expression profiles of all candidates obtained by qRT-PCR corroborated the RNA-Seq data, reinforcing that our in silico analysis reflects the proper expression behavior of DEGs.

Massive transcriptional changes following UV-C radiation have been reported for genes involved in the biosynthesis of phenolic compounds that mediate secondary metabolism in plants, being the majority part of the flavonoids and phenylpropanoids metabolic pathways [17,35,50,69,70,71,92]. These compounds are also involved in metabolic adaptations at the priming phase, during which differential biosynthesis of secondary metabolites leads the plant to a standby state of alertness, being characteristic processes and an important aspect of priming events [19,91]. As expected, the expression of seven selected *A. stenosperma* metaDEGs encoding for enzymes associated with the biosynthesis of alkaloids, flavonoids, and phenylpropanoids (IRL, N8DT2, STS1 and 2, UGT1, BBE, and PAL3) were induced upon UV-C exposure, excepted for UGT2 that was downregulated. Most of these candidates were also induced by other stresses, with only two genes being more activated by drought (N8DT2) or nematode infection (IRL) than by UV exposure. N8DT2 is responsible for the prenylation of the flavonoid naringenin and is involved in pathogen resistance mediated by the MAPK- and SA-dependent activation of NPR1 [93]. Likewise, IRL catalyzes the production of isoflavonoid phytoalexins, which are potent antifungal agents and have recently been associated with maintaining redox homeostasis under stress conditions [94].

UGT genes encode for members of the UDP-Glycosyltransferase superfamily involved in many functions in plants, including the synthesis of anthocyanins and flavonoids. UGTs also participate in the responses to diverse stresses and induction of defense priming during SAR [57,95]. Besides being selected here as *A. stenosperma* metaDEG candidates, three UGTs were listed as commonly multistress-responsive genes by [55] and as one reliable marker of the priming phase by [8].

BBE gene, which displayed the highest induction by UV-C, encodes a flavoprotein that catalyzes diverse reactions in plants, including alkaloid and cannabinoid biosynthesis and alcohol oxidation, and could be involved with immune responses and defense against several pathogens [96]. PAL catalyzes the first step in the phenylpropanoid pathway, whereas STS, downstream of PAL, is responsible for synthesizing the phytoalexin resveratrol, which protects plants against a variety of abiotic and biotic stresses [59]. The strong induction (>139-fold) of the two *Arachis* STS genes following UV-C treatment agrees with our previous studies [21,30]. However, the molecular mechanisms behind their induction in response to UV radiation are still not completely understood. Together, these findings indicate that, as previously observed in other plants, UV-C could regulate the secondary metabolic process in *A. stenosperma* through the biosynthesis and accumulation of flavonoids, resveratrol, alkaloids, and phenylpropanoids. These compounds are crucial to trigger and establish priming defense, protection against phytopathogens, and adaptation to abiotic environmental factors.

### 3.4. UV and Multiple Stresses Alter Cell Wall Dynamics in A. stenosperma

The induction and accumulation of phenolic compounds that mediated the production of secondary metabolites following UV-C exposure might cause important alterations in the cell wall structure, stability, and integrity [71,92]. Moreover, when challenged by stressful conditions, plants protect themselves by altering cell wall properties and remodeling the architecture of its components, being certain changes specific to one type of stress [97]. The plant cell wall is a complex matrix of polysaccharides, structural glycoproteins, and secondary metabolites that dynamically react to pathogen attack and environmental changes and, therefore, emerges as an essential component of defense priming [98,99].

Here, EXLB, encoding for an expansin-like B, and EGC, containing a typical α-expansin domain, were upregulated in response to all four stresses studied. Plant expansins are involved in cell wall loosening and are active components of stress-associated responses, commonly related to defense priming processes [8,57]. Moreover, the upregulation of EXLB and EGC genes is consistent with our recent findings that the overexpression of an *A. duranensis* EXLB gene enhanced tolerance to abiotic and biotic stresses by mediating the induction of a defense primed state [100].

In addition to their involvement in cell wall structure and formation, LAC and PRP emerged as candidate genes to confer resistance/tolerance to different stress conditions [101,102]. In agreement with this potential novel biological role, *A. stenosperma* LAC and PRP genes were highly induced by abiotic stresses (UV exposure and drought) but downregulated in response to biotic stresses (fungal and nematode infection). Likewise, FLA is one of the few candidates downregulated in response to fungus, UV, and drought treatments. FLA encodes a fasciclin-like arabinogalactan protein associated with both structural and signaling function in plant cell walls and, in accordance with our results, was also strongly downregulated during SAR establishment and defense priming [57,60], as well as in response to different distinct multi-stresses conditions [55]. All these transcriptional alterations in genes involved in cell wall dynamics suggest that biotic and abiotic stresses can trigger wall structure and integrity modifications, which may be determinants in *A. stenosperma* adaptation to marginal habitats.

Other genes playing regulatory roles in defense responses to various stress conditions, such as Nudix hydrolase (NUDT2) and syntaxin (SYP121) [103,104], were regulated in response to all studied stresses. In particular, NUDT2, as the FLA gene, has also been identified as involved in defense priming responses during SAR and is commonly modulated by various combined stresses [55,57]. On the other hand, another group of genes, such as eGLC, LBD1, and AKT1, were not responsive to drought stress. Still, the involvement of these defense-associated proteins in response to pathogen attack remains little understood.

## 4. Materials and Methods

### 4.1. Plant Materials and Experimental Design

Seeds of wild diploid *Arachis stenosperma* (accession V10309) were obtained from the Active Germplasm Bank of Embrapa Genetic Resources and Biotechnology (Cenargen, Brasília, Brazil). Seeds were germinated on germitex paper with 2% (*w*/*v*) ethrel (2-chloroethylphosphonic acid) to break dormancy and 0.05% (*w*/*v*) Thiram (tetramethylthiuram disulfide) to prevent fungal contamination. Plantlets were transferred to 500 mL pots containing soil and cultivated under greenhouse conditions for 40 days with monitored pest control, humidity, and fertilization conditions.

Fully expanded quadrifoliate leaves were collected in the afternoon from 15 healthy *A. stenosperma* plants (approximately 20 leaves per individual) for immediate use. Ultraviolet (UV) treatment was carried out based on the methodology previously established by our group for *Arachis* species [30]. In short, 300 detached leaves were randomly distributed in six trays (45 cm × 30 cm) containing a layer of sterilized germitex paper that covered a cotton layer (approximately 1 cm) moistened with 500 mL deionized water. Three trays (technical triplicates) containing 50 leaves each (UV-treated group) were immediately placed at room temperature in a laminar flow chamber (Trox^®^ Model FLV series: 235-81; Curitiba, PR, Brazil) and exposed for 150 min to 3.4 mW/cm^2^ UV-C radiation provided by a UV-C lamp (254 nm; model Philips TUV 30W/G30 T8 Longlife). Parameters for UV-C treatment were based on disinfection conditions previously described for peanuts [105]. Likewise, the other three trays with leaves of non-treated control (CTR) group were maintained in the same conditions but under white light. After UV and CTR treatments, four leaflets of each leaf were separated from the petiole, immediately frozen in liquid nitrogen, and stored at −80 °C for RNA extraction. The experiment was repeated under the same conditions (biological replicate) 20 days later.

### 4.2. RNA Extraction and Library Sequencing

Total RNA was extracted from *A. stenosperma* leaflet samples following a CTAB modified protocol previously described by [21]. Before RNA extraction, samples were pooled in the liquid nitrogen at equal amounts per biological replicate to form UV-treated (UV) and non-treated control (CTR) samples. The extracted RNA was purified using the Invisorb^®^ Spin Plant Mini Kit (Invitek, Berlin, Germany), and its integrity was checked according to [52]. cDNA libraries were prepared with “TruSeq Stranded mRNAseq Sample Prep kit” (Illumina Inc., San Diego, CA, USA) and sequenced at the Roy J. Carver Biotechnology Center (University of Illinois, Urbana, IL, USA) on the Illumina HiSeq2500. Raw reads (paired-end 2 × 250 bp) were trimmed, and the quality was checked as described before [48]. Transcriptomic raw data is available on Sequence Read Archive (NCBI-SRA) database under the BioProject number PRJNA284674.

### 4.3. In silico Expression Profiling

Cleaned high-quality raw reads were quantified into the reference genome of *A. duranensis* (version 1) [44] using the default parameters of Kallisto v0.46.1 [106]. The count table resulting from the analysis was used as input for differential expression analysis by EdgeR [107]. Genes were considered as DEGs (Differentially Expressed Genes) if their relative gene expression levels had an adjusted *p*-value (false discovery rate; FDR) < 0.05 and at least a 4-fold change (FC) value between UV and CTR samples (log2FC > 2 or <−2). Only the genes identified as DEGs were considered for further analyses. The Circos plot depicting the distribution of the DEGs in the *A. duranensis* chromosomes (http://peanutbase.org/ (accessed on 10 October 2021)) was generated using Circa software (http://omgenomics.com/circa/ accessed on 10 October 2021)).

### 4.4. Functional Analysis of A. stenosperma DEGs

*A. duranensis* gene models functional annotation (http://peanutbase.org/ (accessed on 20 September 2021)) was used to assign the *A. stenosperma* DEGs into Gene Ontology (GO) categories. The hypergeometric test implemented in the FUNC package [108] was performed to test for significantly overrepresented GO terms among the DEGs using default parameters, as previously described [28]. Only terms with a family-wise error rate (FWER) < 0.05 for overrepresentation were considered in the analysis.

The functional classification of the DEGs was inferred using the online tool Mercator v3.6 (https://www.plabipd.de/portal/mercator-sequence-annotation (accessed on 15 October 2021)), searching against the *A. thaliana* reference database and SwissProt/UniProt, with the default settings. These results were further submitted to MapMan software (http://mapman.gabipd.org/ (accessed on 15 October 2021)) to visualize the gene expression data in different pathways involved in stress responses. Plant transcription factors (TFs), transcriptional regulators (TRs), and protein kinases (PKs) were then identified from DEGs and classified into gene families by using the *A. duranensis* database of the iTAK program [109]. The expression profiles of *A. stenosperma* DEGs identified as TFs, TRs and PKs were then subjected to heatmap construction in ggplots package [110] using the transcriptome data here obtained (UV exposure) and those previously published by our group from roots of *A. stenosperma* plants submitted to biotic (*M. arenaria* infection) and abiotic (dehydration and dry-down treatments) stresses [28,46,53].

### 4.5. Functional Analysis of A. stenosperma NBS-LRR Gene Family

*A. stenosperma* DEGs belonging to nucleotide-binding site and leucine-rich repeats (NBS-LRR) gene family was identified and classified from the 345 NBS-LRR gene models described for *A. duranensis*, according to [44,48]. The expression profiles of *A. stenosperma* DEGs encoding NBS-LRRs were used to build a heatmap using the gplots package, as described above. The in silico expression behavior of *A. stenosperma* NBS-LRR genes was further validated by quantitative reverse transcription PCR (qRT-PCR) analysis, as described below.

### 4.6. Meta-Analysis of A. stenosperma RNA-Seq Data

A comparative transcriptome meta-analysis of *A. stenosperma* plants submitted to abiotic (UV exposure, dehydration, and dry-down treatments) and biotic (nematode infection) stresses was carried out separately, as recently described [53]. For that, we explored the transcriptome data obtained in the present study (UV exposure) and our previously published RNA-Seq data, comprising a total of ten libraries (Table S3) of *A. stenosperma* plants submitted to these stress treatments [28,46,53]. The genes with an adjusted *p*-value < 0.05 and the same expression trend (up or down regulated) for all comparisons were considered commonly regulated metaDEGs for abiotic or biotic stresses, as described by [53]. The Venn diagram was generated using the ‘Draw Venn Diagram’ webtool (bioinformatics.psb.ugent.be/webtools/Venn/).

### 4.7. Identification of Orthologs of Plant Priming Markers

To find *A. duranensis* orthologs of the 44 universal *A. thaliana* gene markers of plant priming [8], we examined a set of 35,238 protein orthogroups, previously characterized by our team [54]. These groups of putatively orthologous genes (orthogroups) were inferred by the comparative analysis across the predicted proteomes of 22 plant species, including *A. thaliana* and *A. duranensis*, using the OrthoFinder v1 software [111]. The *A. duranensis* orthologs of the plant priming markers were then found by searching the corresponding *A. thaliana* gene ID [8] in this previously characterized set of protein orthogroups [54].

### 4.8. Expression Analysis by qRT-PCR

The same RNA pools (UV and CTR groups) used for cDNA library construction and sequencing were used for the expression profiling of selected DEGs by qRT-PCR analysis. In addition, total RNA extracted from our previously conducted stress treatments was also analyzed by qRT-PCR, comprising *A. stenosperma* roots subjected to 12 days of dry-down treatment [61] and infection with the RKN *M. arenaria* for 3, 6, and 9 days [62] and leaves challenged with *C. personatum* for 24, 48, and 72 h [45]. Genomic DNA contaminants were removed from total RNA (2 µg) by DNAse treatment, and cDNA synthesis was carried out in the same tube as previously described [52] and used as the template for qRT-PCR reactions.

Specific primer pairs for the ten NBS-LRR genes and 18 putative memory genes (Table S2) were designed using previously described parameters [52]. qRT-PCR reactions were conducted in technical duplicates for each sample on a StepOne Plus Real-Time PCR System (Applied Biosystems, Foster City, USA), according to [52]. No template control (NTC) samples were included as negative controls. The online real-time PCR Miner tool [112] was used to estimate the primer efficiency and optimal cycle of quantification (Cq) values. Expression ratios of transcripts from the UV-stressed samples relative to non-stressed control samples (Relative Quantification; RQ) were determined and statistically tested using the REST 2009 v. 2.0.13 software [113] and normalized with two *Arachis* reference genes (60S and GAPDH), in accordance with [114].

## 5. Conclusions

Plants are constantly exposed to recurrent stresses and evolved mechanisms to remember experienced events through an adaptive priming strategy. This phenomenon prepares plants for a faster and stronger response to subsequent stress exposure and is marked by enhanced activation of inherent defense systems. Although the stress tolerance mediated by priming has attracted a growing interest in the scientific community in recent years, the biotechnological application of genes involved in molecular priming strategies has been little explored. The present work describes the transcriptome changes triggered in *A. stenosperma* by UV-C radiation, an elicitor of priming defense responses. *A. stenosperma* was used as a wild species model highly adapted to marginal habitats and harboring stress-resilience traits. Molecular mechanisms underlying defense responses to multiple stresses in this wild species were observed, confirming that the expression of a series of genes encoding transcriptional regulators, kinases, and NBS-LRRs was drastically altered in response to UV-C treatment. Meta-analysis of *A. stenosperma* transcripts responsive to both biotic and abiotic stresses further identified a subset of genes commonly modulated by multiple primary stimuli. These genes are components of the priming fingerprinting in the species and could be part of the molecular activities in the so-called priming phase that prepares plants for enhanced responsiveness upon subsequent challenge. Since priming genes are involved in plant adaptation to stressful conditions, these genes constitute promising candidates for developing genetic markers for breeding and genetic engineering towards improved and more sustainable multistress-tolerant crops. In addition, our results provide a molecular framework for further elucidation of mechanisms behind the establishment of priming defense and stress memory acquisition in wild species.

## Figures and Tables

**Table 1:** Orthogroups sharing *Arachis duranensis* gene models identified as metaDEGs and *Arabidopsis thaliana* universal gene markers [8].

Protein Family (Orthogroup) ^a^	Universal Gene Markers (*Arabidopsis thaliana*)	metaDEGs (*Arachis duranensis* Gene Models)	BLAST
OG0000001	AT4G21390	Aradu.9LI83; Aradu.BTP7B; Aradu.NI7KT	Receptor-like serine/threonine kinase
OG0000057	AT1G07650	Aradu.4W18S; Aradu.KB9NK	Leucine-rich repeat (LRR) receptor-like serine/threonine-protein kinase
OG0000067	AT4G38620	Aradu.CT448; Aradu.18EWZ	MYB-like transcription factor
OG0000286	AT4G02340	Aradu.JJ61J	Epoxide hydrolase
OG0001961	AT5G48380	Aradu.8IX7E	BAK1-interacting receptor-like kinase 1
OG0001992	AT3G62870	Aradu.32KIU; Aradu.9L616	60S ribosomal protein L7A (RPL7aB)
OG0006245	AT2G44500	Aradu.YX17V	O-fucosyltransferase family protein

^a^ Orthogroups code generated by OrthoFinder analysis, according to [54].

## Data Availability

The data used to support the findings of this study are available as on Sequence Read Archive (NCBI-SRA) database under the BioProject number PRJNA284674.

## Supplementary Materials

The following supporting information can be downloaded at: https://www.mdpi.com/article/10.3390/plants11030408/s1, Table S1. Transcriptome analyses information. Illumina HiSeq2500 sequencing data of *Arachis stenosperma* transcriptome of non-treated control (CTR) and UV-C treated (UV) samples and mapping of reads to the reference genome. Table S2. Primers used for qRT-PCR analysis. Table S3. RNA-Seq libraries. *Arachis stenosperma* libraries (RNA-Seq) used in the meta-analysis and their corresponding NCBI accession numbers. Table S4. Orthogroup analysis. Orthogroups sharing *Arachis duranensis* gene models identified as significantly expressed in response to UV and *Arabidopsis thaliana* universal gene markers [8]. The first column represents the orthogroups code generated by OrthoFinder analysis, according to [54]. Table S5. Exclusive cross-stress DEGs. List of 743 “exclusive cross-stress” *Arachis duranensis* gene models coding for DEGs (Differentially Expressed Genes) identified as putatively responsive to concurrent UV-C and wounding stresses in *Arachis stenosperma*.

## Abbreviations

DAI	Days after Inoculation
DEG	Differentially Expressed Gene
FC	Fold Change
FDR	False Discovery Rate
GO	Gene Ontology
HAI	Hours after Inoculation
J2	Second-stage Juveniles
JA	Jasmonic Acid
PK	Protein Kinase
qRT-PCR	quantitative Reverse Transcription-olymerase Chain Reaction
RKN	Root-Knot Nematode
ROS	Reactive Oxygen Species
SA	Salicylic Acid
SAR	Systemic Acquired Resistance
TF	Transcription Factor
TR	Transcriptional Regulator
UV	Ultraviolet
