# Supplementary material for: Transcriptome Responses of Wild Arachis to UV-C Exposure Reveal Genes Involved in General Plant Defense and Priming
Source: Plants (Basel). 2022 Feb 2;11(3):408. doi: 10.3390/plants11030408 (PMC8838480; doi:10.3390/plants11030408)
Supplement: Supplementary file 1 [file plants-11-00408-s001.zip › plants-1515800-supplementary.pdf]

Supplementary Materials

Transcriptome responses of wild *Arachis* to UV-C exposure reveal genes involved in general plant defense and priming

Andressa Cunha Quintana Martins; Ana Paula Zotta Mota; Paula Andrea Sampaio Vasconcelos Carvalho; Mário Alfredo Saraiva Passos; Marcos Aparecido Gimenes; Patricia Messenberg Guimaraes; Ana Cristina Miranda Brasileiro*

*** Corresponding author:** ana.brasileiro@embrapa.br

Table S1. Transcriptome analyses information. Illumina HiSeq2500 sequencing data of *Arachis stenosperma* transcriptome of non-treated control (CTR) and UV-C treated (UV) samples and mapping of reads to the reference genome.

Libraries	CTR	UV
**Total number of raw reads**	36,001,334	38,886,104
**Number of quantified reads into the reference** [a]	27,623,900	28,063,800
**Average number of gene models**	24,113	24,978
Number of significantly expressed genes [b]	13,858	
Number of DEGs [c] with log2FC > 2 or < -2	5,751 (2,679/3,072 [d])	
Number of DEGs [c] with log2FC > 3 or < -3	3,215 (1,574/1,641 [d])	
Number of DEGs [c] with log2FC > 4 or < -4	1,809 (968/841 [d])	

[a] *Arachis duranensis* reference genome (version 1) (Bertioli *et al*. 2016) available at (http://peanutbase.org/).

[b] FDR< 0.05.

[c] Differentially Expressed Genes (DEGs) between UV and CTR samples.

[d] Upregulated/downregulated DEGs.

Table S2. Primers used for qRT-PCR analysis.

Primer name	Putative function	Forward primer (5'-3')	Reverse primer (5'-3')	Amplicon (bp)	Efficiency of primer	Reference
**Secondary metabolism**						
IRL	Isoflavone reductase homolog	GAAAGCAGCTCCACAAGACC	CCATGCGTGTGTAGTTGACC	195	0,8679	This study.
STS1	Stilbene Synthase	AAGGCCATCAAGGAATGGGG	ATGGGTCGAGGCCTAAGAGT	124	0,8595	This study.
STS2	Stilbene Synthase	CAATTGGCACGGCAAATCCA	ACGATGGTGCCTTGTATGCA	210	0,8354	This study.
UGT1	7-deoxyloganetin glucosyltransferase	TGAACTCAGAAGGCAACACG	TGAACTTTGGCAGCATAACG	144	0,8714	This study.
UGT2	UDP-Glycosyltransferase	TCCGTTGGTGGCTTTTTATC	CTGTCCACCACCTTCTTGGT	182	0,8528	This study.
BBE	FAD-binding Berberine family protein	TTGGTGAAGTGGGATGTGAA	ATAGGTGGCCCTTGGAGAGT	114	0,9096	This study.
N8DT2	Naringenin 8-dimethylallyltransferase 2	GCCAGTACGTTTGGGTCCTA	AAGAGAAGCGCGATACCAGA	172	0,8478	This study.
PAL3	Phenylalanine ammonia-lyase class 3	TGGGGTTAATTTCTGCTTGG	GATCCAAACAAATCCGTGCT	187	0,875	This study.
**Cell wall**						
EXLB	Expansin like-B	TGGGTTCTCAACATCAAACACT	CGCAGCTACAACCCACACTA	150	0,8449	Brasileiro et al., 2015.
EGC	EG45-like domain containing protein	GTTCCCATCGAGCAATCTGT	GGCAGCGTAATCAACGATCT	160	0,8708	This study.
FLA	Fasciclin like-Arabinogalactan	GGGTTCTAACAAGGCTGCTG	CCTTGGTTGAGGGAGTTCAA	154	0,8628	This study.
LAC	Laccase	AACCGTATGCAACCTTCCAG	GTGGAGGTTGACTTGGGAAA	185	0,8516	This study.
PRP	14 kDa proline-rich protein	TTCCAAAACCACTTCCCTTG	ATTGTGCTTTGGTGGAGGAG	103	0,8948	This study.
**Basal defense**						
eGLC	Glucan endo-1,3-beta glucosidase	TGGGTGTATGCTATGGTGGA	GGATTGGAGTTGGTCATTGG	182	0,8772	This study.
NUDT2	Nudix hydrolase 2-like	CGGTGAATGAAGGTGAGGAT	GTTGCAACAAGCAAGCAAAG	156	0,8715	This study.
SYP121	Syntaxin-121	GAACAGCTCGACGACATTGA	GCCAAGGCGATTATGAGAAG	152	0,8383	This study.
**Stress signal transduction**						
LBD1	LOB domain-containing protein 1	TGGCAAAAACACAAGCTGAG	GAGGGCTTGGAACATTGAAA	168	0,8869	This study.
AKT1	Potassium channel AKT1	AAGAACGGTTGGACACCAAG	TGCTCTCTTGGGGTAGAGGA	190	0,8788	This study.
**R-genes (NBS-TIR)**						
NBS36	Disease resistance protein	GAGCTGCAAAACATCGTTGA	TTGGACTTTCCCCTTCTCCT	162	0,9009	Mota et al., 2018.
NBS29	Disease resistance protein	GTGCTTGCGAAATATGCTCA	CATGATCCACCACCCTCTCT	154	0,8700	This study.
NBS47	Disease resistance protein	TCACCTTTATGCTGCACTGG	TCAACGATGTTTTGCAGCTC	192	0,9008	Mota et al., 2018.
NBS34	Disease resistance protein	GGGACAAAATTCCATTGTGG	CACATCACCGACAATTTTGC	154	0,8864	Mota et al., 2018.
NBS33	Disease resistance protein	GCTTTCCGAGAACACGATTC	CGCTACCAGCAAGAAACACA	158	0,8676	Mota et al., 2018.
NBS27	Disease resistance protein	TCAAAGCTCAATCCCCAAAC	TTGAGTCGGAAAAAGGTGCT	185	0,9000	This study.
NBS39	Disease resistance protein	TGGTCCAGAGCCACATAACA	ACAAAACGGATGGATGAAGC	183	0,8800	This study.
**R-genes (NBS-CC)**						
NBS40	Disease resistance protein	TGATTATGGAGGGCGGTTAG	CGTGCTCTTTCCAGATCCTC	181	0,8828	Mota et al., 2018.
NBS44	Disease resistance protein	GTGGCAAGATCCTCGTTCTC	CCTCCTTCACATCCCTCGTA	156	0,8750	Mota et al., 2018.
NBS20	Disease resistance protein	CCAAAGGGCTACCAATTCAA	CCAGAACCATAAGGCTCCAA	155	0,8774	This study.

Table S3. RNA-Seq libraries. *Arachis stenosperma* libraries (RNA-Seq) used in the meta-analysis and their corresponding NCBI accession numbers.

Accession	Library	Type of stress	Time-point [a]	Total reads	Reference
SAMN03734821	*Meloidogyne arenaria* inoculation	Biotic	0 DAI (control)	6,651,071	Guimaraes et al., 2015
SAMN03734822	*Meloidogyne arenaria* inoculation		3 DAI	7,162,500	Guimaraes et al., 2015
SAMN03734823	*Meloidogyne arenaria* inoculation		6 DAI	6,333,182	Guimaraes et al., 2015
SAMN03734824	*Meloidogyne arenaria* inoculation		9 DAI	6,202,969	Guimaraes et al., 2015
To be submitted	UV-C exposure	Abiotic	0 DAI (control)	36,001,334	This study
To be submitted	UV-C exposure		150 minutes	38,886,104	This study
SAMN08225801	Drought (Dehydration)		0 minutes (control)	12,219,081	Vinson et al., 2018
SAMN08225802	Drought (Dehydration)		25 - 150 minutes	12,587,737	Vinson et al., 2018
SAMN16049051	Drought (Dry-down)		0 DAI (control)	100,326,990	Mota et al., 2021
SAMN16049053	Drought (Dry-down)		7 DAI	112,787,062	Mota et al., 2021

[a] DAI = Days after Inoculation.

Table S4. Orthogroup analysis. Orthogroups sharing *Arachis duranensis* gene models identified as significantly expressed in response to UV and *Arabidopsis thaliana* universal gene markers (Baccelli et al. 2019). The first column represents the orthogroups code generated by OrthoFinder analysis, according to Mota et al. 2020.

Protein family (orthogroup)	*A. thaliana* universal gene markers	Number of orthologs	*A. duranensis* gene models	Annotation (Baccelli et al., 2019)
OG0000001	AT4G21390	49	Aradu.039A1; Aradu.15JH1; Aradu.1H2D4; Aradu.25UEB; Aradu.2D3LB; Aradu.494EG; Aradu.4HH0P; Aradu.56PKD; Aradu.5MH7X; Aradu.5P7RM; Aradu.80RUZ; Aradu.853ZC; Aradu.8QX2U; Aradu.9LI83; Aradu.A6YGJ; Aradu.ACY83; Aradu.AX3Z0; Aradu.B17TN; Aradu.BI4ZP; Aradu.BTP7B; Aradu.CED81; Aradu.CS1K3; Aradu.D06PB; Aradu.D0NVI; Aradu.D6B0G; Aradu.EHP84; Aradu.EP9MB; Aradu.EZG2L; Aradu.FC0YK; Aradu.G2KK1; Aradu.HB34E; Aradu.IJE02; Aradu.IR1JE; Aradu.IW9Q8; Aradu.JTL52; Aradu.L4EY2; Aradu.L65YC; Aradu.LF5V6; Aradu.LR05I; Aradu.LW36F; Aradu.N1UIU; Aradu.NI7KT; Aradu.P08HB; Aradu.R8UZU; Aradu.S4DK6; Aradu.X00D6; Aradu.YVF29; Aradu.YW23C; Aradu.Z4BE5	S-locus lectin protein kinase family protein
OG0000042	AT5G42020	24	Aradu.0J1NB; Aradu.2IN3E; Aradu.66JGA; Aradu.938IA; Aradu.9GM08; Aradu.DLG8U; Aradu.EXN4Y; Aradu.EXU0Y; Aradu.G6ZA5; Aradu.GBS9S; Aradu.L2DZ2; Aradu.L8IJK; Aradu.LMA8E; Aradu.M35Z5; Aradu.MS98X; Aradu.QCH7F; Aradu.QL2WJ; Aradu.R9QAM; Aradu.RER3E; Aradu.S6N02; Aradu.SB9IZ; Aradu.T50NW; Aradu.TET8U; Aradu.Z1N61	Heat shock protein 70 (Hsp 70); BIP2; ATP binding
OG0000057	AT1G07650	14	Aradu.29VTX; Aradu.3IJ3D; Aradu.3N4PQ; Aradu.4W18S; Aradu.7V7LG; Aradu.98U3Z; Aradu.AG5I3; Aradu.E5HJY; Aradu.FQT6J; Aradu.KB9NK; Aradu.Q3GPP; Aradu.WZF00; Aradu.X8I5Q; Aradu.Y37EJ	leucine-rich repeat transmembrane protein kinase, putative
OG0000067	AT4G38620	11	Aradu.0Z09C; Aradu.18EWZ; Aradu.2Z52F; Aradu.57USE; Aradu.862GA; Aradu.90QVH; Aradu.AB7NH; Aradu.CT448; Aradu.GLV35; Aradu.GN6SE; Aradu.T2ULI	MYB4
OG0000236	AT4G39330	8	Aradu.DF39S; Aradu.GP6IB; Aradu.LV8M3; Aradu.MI2LX; Aradu.V8KPM; Aradu.KX25J; Aradu.PV1T4; Aradu.V4LAJ	CAD9 (cinnamyl alcohol dehydrogenase 9)
OG0000286	AT4G02340	8	Aradu.BAW8N; Aradu.D8HQK; Aradu.E26HT; Aradu.H83ZS; Aradu.JJ61J; Aradu.X74QT; Aradu.YJ394; Aradu.ZL56D	epoxide hydrolase, putative

Protein family (orthogroup)	*A. thaliana* universal gene markers	Number of orthologs	*A. duranensis* gene models	Annotation (Baccelli et al., 2019)
OG0000287	AT1G54730	6	Aradu.3RL8Y; Aradu.55RDX; Aradu.DQM3T; Aradu.MI3UP; Aradu.RRR8S; Aradu.Z5IUC	sugar transporter
OG0000290	AT3G12620	6	Aradu.289R9; Aradu.DB4LT; Aradu.EIY0H; Aradu.JA809; Aradu.U7Z4S; Aradu.T482L	protein phosphatase 2C family protein
OG0000558	AT5G23750	6	Aradu.3G0W0; Aradu.5I6E9; Aradu.BSP9G; Aradu.EN7BC; Aradu.PQX4Z; Aradu.ZV7LR	remorin family protein
OG0000615	AT5G19440	5	Aradu.2Q9YT; Aradu.3R43N; Aradu.B2GQ6; Aradu.JNZ5A; Aradu.NA6VL	cinnamyl-alcohol dehydrogenase, putative
OG0000617	AT2G25737	5	Aradu.JH5R4; Aradu.QR8GU; Aradu.X3HVV; Aradu.IR4I0; Aradu.SG50D	Sulfite exporter TauE/SafE family protein
OG0000675	AT5G16110	5	Aradu.4FV7T; Aradu.6ZE3A; Aradu.D79UM; Aradu.N741F; Aradu.ZJ194	Unknown protein
OG0000710	AT2G18660	5	Aradu.1EH3D; Aradu.6X6HU; Aradu.J3PLI; Aradu.EN5VX; Aradu.SZ6Y0	expansin-like b3 precursor
OG0000772	AT5G03530	5	Aradu.0ZE66; Aradu.8L0RK; Aradu.9M1GG; Aradu.P4R7R; Aradu.VTP3R	RAB GTPase homolog C2a
OG0001069	AT3G23600	4	Aradu.34UTA; Aradu.8Y6AX; Aradu.KN7EE; Aradu.IHP1V	dienelactone hydrolase family protein
OG0001473	AT5G12890	4	Aradu.JX29L; Aradu.71JL3; Aradu.CLU1K; Aradu.T8G13	UDP-glucoronosyl/UDP-glucosyl transferase family protein
OG0001760	AT5G39950	3	Aradu.4V2X0; Aradu.3UE4N; Aradu.VS3UG	thioredoxin 2
OG0001876	AT1G53910	3	Aradu.00XL9; Aradu.KXA2K; Aradu.R23AB	RAP2.12; DNA binding / transcription factor (ethylene response factor 74)
OG0001961	AT5G48380	2	Aradu.8IX7E; Aradu.JXB2I	BAK1-interacting receptor-like kinase 1, BIR1
OG0001992	AT3G62870	2	Aradu.32KIU; Aradu.9L616	60S ribosomal protein L7A (RPL7aB)
OG0002075	AT1G70160	2	Aradu.963L7; Aradu.7F7LP	Zinc finger MYND domain protein
OG0002117	AT3G26935	2	Aradu.053WE; Aradu.NY77B	zinc finger (DHHC type) family protein
OG0002906	AT5G59350	2	Aradu.R7YU5; Aradu.13SDX	Unknown protein

Protein family (orthogroup)	*A. thaliana* universal gene markers	Number of orthologs	*A. duranensis* gene models	Annotation (Baccelli et al., 2019)
OG0002922	AT2G38740	2	Aradu.J2HBF; Aradu.U6HMB	haloacid dehalogenase-like hydrolase family protein
OG0004013	AT5G63140	2	Aradu.PZB3C; Aradu.VM6LR	Purple acid phosphatase 29
OG0004037	AT5G45130	2	Aradu.9CH46; Aradu.S4DUV	RAB HOMOLOG 1; GTP binding
OG0004205	AT1G13340	2	Aradu.0RG1D; Aradu.I8Q2P	IST1-LIKE 6
OG0004300	AT1G21750	2	Aradu.9645F; Aradu.IT29W	ATPDIL1–1 (PDI-LIKE 1–1); protein disulfide isomerase
OG0004657	AT5G64813	1	Aradu.8I2Q1	LIP1 (Light Insensitive Period1); GTPase
OG0005045	AT3G52050	1	Aradu.U7Z89	5′-3′ exonuclease family protein
OG0005835	AT5G62790	1	Aradu.NH17S	DXR (1-deoxy-d-xylulose 5-phosphate reductoisomerase)
OG0006245	AT2G44500	1	Aradu.YX17V	O-fucosyltransferase family protein
OG0007816	AT3G33530	1	Aradu.I6QB3	transducin family protein /WD-40 repeat family protein
OG0008263	AT4G05180	1	Aradu.Y8LHL	Photosystem II subunit Q (PSBQ-2)
OG0008284	AT1G63900	1	Aradu.CD5BK	zinc finger (C3HC4-type RING finger) family protein
OG0009489	AT4G02790	1	Aradu.39R56	GTP-binding family protein
OG0012593	AT3G09580	1	Aradu.CMM2K	amine oxidase family protein

Table S5. Exclusive cross-stress DEGs. List of 743 "exclusive cross-stress" *Arachis duranensis* gene models coding for DEGs (Differentially Expressed Genes) identified as putatively responsive to concurrent UV-C and wounding stresses in *Arachis stenosperma*.

№	Gene model	Annotation
1	Aradu.01T8N	receptor-like protein kinase 2
2	Aradu.02TGY	uncharacterized protein LOC100803315 [Glycine max]
3	Aradu.02YQS	Disease resistance protein (TIR-NBS-LRR class) family
4	Aradu.039A1	receptor-like serine
5	Aradu.03ENG	Non-specific lipid-transfer protein, putative
6	Aradu.06H1M	receptor-like protein kinase 2
7	Aradu.083WP	Protein kinase superfamily protein
8	Aradu.09F0B	cytochrome c biogenesis protein family
9	Aradu.0CM87	calmodulin-binding family protein
10	Aradu.0G778	unknown protein
11	Aradu.0GB26	sugar transporter protein 7
12	Aradu.0GQ0X	Ribulose-1,5 bisphosphate carboxylase
13	Aradu.0JC72	MATE efflux family protein
14	Aradu.0KX1J	peptide transporter 5
15	Aradu.0MP9W	ATP binding
16	Aradu.0Q24I	Calmodulin binding protein-like
17	Aradu.0Q6Y1	disease resistance protein (TIR-NBS-LRR class), putative
18	Aradu.0QE03	disease resistance protein (TIR-NBS-LRR class), putative
19	Aradu.0QP9J	probable peptide
20	Aradu.0XA87	arogenate dehydratase 6
21	Aradu.0Z2ZN	myb transcription factor
22	Aradu.109YL	Cytochrome P450 superfamily protein
23	Aradu.10ESY	alpha
24	Aradu.11776	Cell wall protein EXP2 n
25	Aradu.144RM	receptor-like serine
26	Aradu.151MH	disease resistance protein (TIR-NBS-LRR class), putative
27	Aradu.152KT	disease resistance protein (TIR-NBS-LRR class), putative
28	Aradu.17BB2	heat shock transcription factor B4
29	Aradu.17JE2	DNA binding protein n
30	Aradu.1AZ9I	cysteine-rich TM module stress tolerance protein
31	Aradu.1C9UI	2-oxoglutarate (2OG) and Fe(II)-dependent oxygenase superfamily protein
32	Aradu.1F2UP	Flavin-binding monooxygenase family protein
33	Aradu.1GZ32	nodulin MtN21
34	Aradu.1H2D4	receptor-like serine
35	Aradu.1M2X1	chlorophyll A
36	Aradu.1T3UD	Bifunctional inhibitor
37	Aradu.1V1PL	protein kinase 1B
38	Aradu.1WD61	Cytochrome P450 superfamily protein
39	Aradu.1ZX0E	dehydration-responsive element-binding protein 3-like [Glycine max]
40	Aradu.200SM	receptor-like protein kinase 1
41	Aradu.20TYF	WRKY family transcription factor
42	Aradu.212W8	Protein kinase superfamily protein
43	Aradu.228PS	receptor-like protein kinase 2
44	Aradu.259PC	WRKY family transcription factor
45	Aradu.25UEB	receptor kinase 2
46	Aradu.26P3H	receptor-like protein kinase 2
47	Aradu.27C7S	beta-fructofuranosidase
48	Aradu.27DYI	MLO-like protein 8-like [Glycine max]
49	Aradu.28ASR	ATP binding
50	Aradu.28WLZ	Cell wall protein EXP2 n

№	Gene model	Annotation
51	Aradu.2C43K	Glutathione S-transferase family protein
52	Aradu.2GH9Y	glutamate decarboxylase
53	Aradu.2J4YI	protein YLS7-like [Glycine max]
54	Aradu.2K11I	heat shock transcription factor A3
55	Aradu.2KV4N	pfkB-like carbohydrate kinase family protein
56	Aradu.2ML72	Cytochrome P450 superfamily protein
57	Aradu.2Q1S0	receptor kinase 1
58	Aradu.2RV29	glycerol-3-phosphate acyltransferase 2
59	Aradu.2TG90	Glutathione S-transferase family protein
60	Aradu.2UI08	Protein kinase superfamily protein
61	Aradu.2V4EQ	WRKY family transcription factor
62	Aradu.2W8YR	Cytochrome P450 superfamily protein
63	Aradu.2Y4F9	Cytochrome P450 superfamily protein
64	Aradu.2Z92T	Core-2
65	Aradu.32QV2	Cytochrome P450 superfamily protein
66	Aradu.32RQ0	RING-H2 finger protein 2B
67	Aradu.346IW	putative pectinesterase
68	Aradu.3602N	Glutathione S-transferase family protein
69	Aradu.37G4U	Small nuclear ribonucleoprotein family protein
70	Aradu.383XS	calcium-dependent protein kinase 32
71	Aradu.38L67	MATE efflux family protein
72	Aradu.38Y8J	calcium-transporting ATPase 4, plasma membrane-type protein
73	Aradu.398HV	Cytochrome P450 superfamily protein
74	Aradu.39HJQ	SET domain-containing protein
75	Aradu.3A47Y	receptor kinase 1
76	Aradu.3C1R2	Peroxidase superfamily protein
77	Aradu.3CD7I	sugar transporter protein 7
78	Aradu.3GT7T	disease resistance protein (TIR-NBS-LRR class), putative
79	Aradu.3K9KE	Cytochrome P450 superfamily protein
80	Aradu.3S60E	glyceraldehyde-3-phosphate dehydrogenase C2
81	Aradu.3T2TK	GTP-binding nuclear Ran-like protein
82	Aradu.3W0JX	tyrosine aminotransferase 3
83	Aradu.405P3	galactoside 2-alpha-L-fucosyltransferase-like protein
84	Aradu.4262U	Cytochrome P450 superfamily protein
85	Aradu.427SB	Cytochrome P450 superfamily protein
86	Aradu.43J56	zinc finger protein CONSTANS-LIKE 2 [Glycine max]
87	Aradu.43LTP	fatty acyl-CoA reductase 3-like [Glycine max]
88	Aradu.45FY8	Cytochrome P450 superfamily protein
89	Aradu.46DEV	glutamate decarboxylase
90	Aradu.470B5	ethylene-responsive transcription factor 5-like [Glycine max]
91	Aradu.47T2G	Potassium transporter family protein
92	Aradu.494EG	receptor-like serine
93	Aradu.49PAS	Cytochrome P450 superfamily protein
94	Aradu.4B6GH	Cytochrome P450 superfamily protein
95	Aradu.4F030	serine carboxypeptidase-like 17
96	Aradu.4I7WA	xyloglucan endotransglucosylase
97	Aradu.4KE49	disease resistance protein (TIR-NBS-LRR class), putative
98	Aradu.4QE7C	fructose-bisphosphate aldolase 2
99	Aradu.4RE2S	12-oxophytodienoate reductase 2
100	Aradu.4T5J3	Cytochrome P450 superfamily protein
101	Aradu.4X7WG	disease resistance protein (TIR-NBS-LRR class), putative
102	Aradu.4Z2QP	putative phospholipid-transporting ATPase 9-like isoform X1 [Glycine max]
103	Aradu.4Z3ES	Protein kinase superfamily protein

№	Gene model	Annotation
104	Aradu.50M8D	zinc finger CCCH domain protein, putative
105	Aradu.5154U	putative phospholipid-transporting ATPase 4-like isoform X3 [Glycine max]
106	Aradu.5474V	glucan endo-1,3-beta-glucosidase-like [Glycine max]
107	Aradu.54VLY	receptor-like protein kinase 5-like [Glycine max]
108	Aradu.55VHH	Cytochrome P450 superfamily protein
109	Aradu.56PKD	receptor-like serine
110	Aradu.56PSF	Tetratricopeptide repeat (TPR)-like superfamily protein
111	Aradu.58K6Q	receptor-like protein kinase 1
112	Aradu.59NQ7	zinc finger CCCH domain protein
113	Aradu.5CR2H	acetyltransferase (GNAT) domain protein
114	Aradu.5K7C2	protein YLS7 [Glycine max]
115	Aradu.5N0SG	disease resistance protein (TIR-NBS-LRR class), putative
116	Aradu.5P7KT	LETM1-like protein
117	Aradu.5VY9W	receptor-like protein kinase 1
118	Aradu.5Y4LG	FAD-binding Berberine family protein
119	Aradu.5ZC0V	12-oxophytodienoate reductase 2
120	Aradu.60CNJ	disease resistance protein (TIR-NBS-LRR class), putative
121	Aradu.60HCE	catalase 2
122	Aradu.60IB6	Protein kinase superfamily protein
123	Aradu.618TJ	disease resistance protein (TIR-NBS-LRR class), putative
124	Aradu.623Y7	serine acetyltransferase 1
125	Aradu.629Q2	sucrose transporter 2
126	Aradu.62DXS	myb transcription factor
127	Aradu.63K76	peptide transporter 1
128	Aradu.63SET	glucan endo-1,3-beta-glucosidase 11-like [Glycine max]
129	Aradu.64FTH	plasma membrane intrinsic protein 2
130	Aradu.66564	uncharacterized protein LOC102669280 [Glycine max]
131	Aradu.66JGA	heat shock protein 70
132	Aradu.6A07N	Serine protease inhibitor, potato inhibitor I-type family protein
133	Aradu.6AI81	plasma membrane intrinsic protein 2
134	Aradu.6EF8P	receptor kinase 2
135	Aradu.6FA26	receptor kinase 3
136	Aradu.6M14N	UDP-Glycosyltransferase superfamily protein
137	Aradu.6M7C0	receptor-like protein kinase 4
138	Aradu.6N08G	N-(5-phosphoribosyl)anthranilate isomerase
139	Aradu.6NU8A	glutathione reductase
140	Aradu.6R3YG	disease resistance protein (TIR-NBS-LRR class), putative
141	Aradu.6R69J	Cytochrome P450 superfamily protein
142	Aradu.6T93Q	uncharacterized protein LOC102669532 isoform X3 [Glycine max]
143	Aradu.6V6HA	tetraspanin-8-like [Glycine max]
144	Aradu.6W466	NAD(P)-binding Rossmann-fold superfamily protein
145	Aradu.6X9VQ	UDP-glycosyltransferase 74 F1
146	Aradu.6Y8G9	nudix hydrolase homolog 17
147	Aradu.6ZM4I	RING
148	Aradu.73RKR	Indole-3-acetic acid-induced protein ARG2, putative n
149	Aradu.75TKT	disease resistance protein (TIR-NBS-LRR class), putative
150	Aradu.75VJN	Disease resistance protein (TIR-NBS-LRR class)
151	Aradu.76Z6I	LRR receptor-like kinase
152	Aradu.77XML	Chitinase family protein
153	Aradu.786V9	Protein of unknown function (DUF506)
154	Aradu.78F4Z	receptor serine
155	Aradu.78FH9	transmembrane amino acid transporter family protein
156	Aradu.78G42	Glycolipid transfer protein (GLTP) family protein

№	Gene model	Annotation
157	Aradu.78I1X	subtilisin-like serine protease 2
158	Aradu.7A6EX	polyamine oxidase 4
159	Aradu.7DE4K	P-loop containing nucleoside triphosphate hydrolases superfamily protein
160	Aradu.7GF98	uncharacterized protein At2g24330-like isoform X1 [Glycine max]
161	Aradu.7K9KV	ferredoxin 3
162	Aradu.7L2S4	metalloendoproteinase 1-like [Glycine max]
163	Aradu.7M2ZA	unknown protein
164	Aradu.7MF1E	Water-selective transport intrinsic membrane protein 1 n
165	Aradu.7N61Y	plasma membrane intrinsic protein 1
166	Aradu.7R2WH	WRKY family transcription factor
167	Aradu.7SN56	gibberellin 20 oxidase 2-like [Glycine max]
168	Aradu.7TS1N	receptor-like protein kinase 4
169	Aradu.7XS8V	Peroxidase superfamily protein
170	Aradu.80TQM	Glutathione S-transferase family protein
171	Aradu.817IP	receptor-like protein kinase 2
172	Aradu.8203M	DEAD-box ATP-dependent RNA helicase-like protein
173	Aradu.82M1H	Protein kinase superfamily protein
174	Aradu.83N8C	transcription factor bHLH25-like [Glycine max]
175	Aradu.853ZC	receptor-like serine
176	Aradu.88QB9	basic 7S globulin [Glycine max]
177	Aradu.89624	MATE efflux family protein
178	Aradu.8CL43	glutathione S-transferase F4
179	Aradu.8CV4T	Serine protease inhibitor, potato inhibitor I-type family protein
180	Aradu.8D60D	aldose 1-epimerase-like [Glycine max]
181	Aradu.8E2VW	Auxin-responsive protein n
182	Aradu.8EQ04	uncharacterized protein LOC100800538 [Glycine max]
183	Aradu.8J00F	Cytochrome P450 superfamily protein
184	Aradu.8K8TN	plasma membrane intrinsic protein 1
185	Aradu.8LX7K	UDP-Glycosyltransferase superfamily protein
186	Aradu.8MB2V	beta-fructofuranosidase
187	Aradu.8PB60	catalase 2
188	Aradu.8PJ2J	sugar porter (SP) family MFS transporter
189	Aradu.8PM8C	squamosa promoter binding protein-like 1
190	Aradu.8Q6IV	glucan endo-1,3-beta-glucosidase-like [Glycine max]
191	Aradu.8QI9R	type I inositol 1,4,5-trisphosphate 5-phosphatase 11-like [Glycine max]
192	Aradu.8RX2Y	Late embryogenesis abundant (LEA) hydroxyproline-rich glycoprotein family
193	Aradu.8S4TL	Protein phosphatase 2C family protein
194	Aradu.8U98A	ethylene-responsive transcription factor 3 [Glycine max]
195	Aradu.8UK33	Protein kinase superfamily protein
196	Aradu.8V97A	myb transcription factor
197	Aradu.8Y6WE	Actin cross-linking protein
198	Aradu.901R7	Water-selective transport intrinsic membrane protein 1 n
199	Aradu.938IA	heat shock protein 70
200	Aradu.93KPA	probable pectinesterase
201	Aradu.93RCN	protein kinase 2B
202	Aradu.947SP	Peroxidase superfamily protein
203	Aradu.96S2E	glucan endo-1,3-beta-glucosidase 14-like [Glycine max]
204	Aradu.97AME	disease resistance protein (TIR-NBS-LRR class), putative
205	Aradu.97ZLB	DnaJ heat shock amine-terminal domain protein
206	Aradu.98KHN	Protein kinase superfamily protein
207	Aradu.99WG9	Cytochrome P450 superfamily protein
208	Aradu.9EZ7Z	sugar transporter 1
209	Aradu.9G6FH	vacuolar cation
210	Aradu.9I2CB	GTP-binding nuclear protein Ran-3-like [Glycine max]

№	Gene model	Annotation
211	Aradu.9IX37	serine acetyltransferase 2
212	Aradu.9LI83	receptor-like serine
213	Aradu.9SB6V	GTP-binding nuclear protein Ran-3 [Glycine max]
214	Aradu.9SJ9X	ferredoxin 1
215	Aradu.9UW5A	Cytochrome P450 superfamily protein
216	Aradu.9V11P	blue copper protein-like [Glycine max]
217	Aradu.A1LMI	Cytochrome P450 superfamily protein
218	Aradu.A3N3V	glyceraldehyde-3-phosphate dehydrogenase C2
219	Aradu.A47FP	null
220	Aradu.A6YGJ	receptor-like serine
221	Aradu.A9RVD	ADP,ATP carrier protein 1, mitochondrial-like [Glycine max]
222	Aradu.AB7NH	myb transcription factor
223	Aradu.ACY83	receptor-like serine
224	Aradu.AFL8M	Protein kinase superfamily protein
225	Aradu.AH39E	receptor-like protein kinase 2
226	Aradu.AH5QJ	fructose-bisphosphate aldolase 2
227	Aradu.AI2M5	fatty acyl-CoA reductase 3-like [Glycine max]
228	Aradu.AI3EU	receptor-like protein kinase 1
229	Aradu.AJZ98	guanine nucleotide-binding protein alpha-2 subunit isoform X3 [Glycine max]
230	Aradu.ALL9T	Protein of unknown function, DUF538
231	Aradu.AR0PR	Cytochrome P450 superfamily protein
232	Aradu.AS232	lipoxygenase 3
233	Aradu.AT974	myb transcription factor
234	Aradu.AVR14	Cytochrome P450 superfamily protein
235	Aradu.AW9GY	Bifunctional inhibitor
236	Aradu.AX3Z0	receptor-like serine
237	Aradu.B17TN	receptor-like serine
238	Aradu.B18E9	Protein kinase superfamily protein
239	Aradu.B1RD2	disease resistance protein (TIR-NBS-LRR class), putative
240	Aradu.B2SVJ	ATP-binding
241	Aradu.B4D3A	U-box domain-containing protein 19-like [Glycine max]
242	Aradu.B4JF5	Cytochrome P450 superfamily protein
243	Aradu.B561I	60S ribosomal protein L10 [Glycine max]
244	Aradu.B5TNQ	glutaredoxin-C9-like [Glycine max]
245	Aradu.B6CZV	protein YLS7-like [Glycine max]
246	Aradu.B8LPK	Cytochrome P450 superfamily protein
247	Aradu.B90GQ	ethylene-responsive transcription factor 1B
248	Aradu.B9QD5	receptor-like serine
249	Aradu.BB4JP	WRKY family transcription factor
250	Aradu.BH13B	Glycosyltransferase family 61 protein
251	Aradu.BI4ZP	receptor-like serine
252	Aradu.BIM74	scarecrow-like protein 14-like [Glycine max]
253	Aradu.BJ9KH	receptor-like protein kinase 4
254	Aradu.BU4F4	transcription factor RADIALIS-like [Glycine max]
255	Aradu.BX1CC	null
256	Aradu.BXL7K	cysteine-rich TM module stress tolerance protein
257	Aradu.BY4US	disease resistance protein (TIR-NBS-LRR class), putative
258	Aradu.BYT1F	Cytochrome P450 superfamily protein
259	Aradu.BZ66H	serine carboxypeptidase-like 7
260	Aradu.C0QT1	aldehyde dehydrogenase family 2 member C4-like [Glycine max]
261	Aradu.C1KJF	receptor-like protein kinase 2
262	Aradu.C2XPI	UDP-Glycosyltransferase superfamily protein
263	Aradu.C4BQN	Cytochrome P450 superfamily protein
264	Aradu.C4IJ9	UDP-Glycosyltransferase superfamily protein
265	Aradu.C64A0	receptor-like protein kinase 2

№	Gene model	Annotation
266	Aradu.C87QH	ethylene-responsive transcription factor 1B
267	Aradu.C90G3	WRKY family transcription factor
268	Aradu.C9QSM	disease resistance protein (TIR-NBS-LRR class), putative
269	Aradu.CCH3U	receptor-like protein kinase 2
270	Aradu.CDX53	Phosphatidylinositol 3- and 4-kinase family protein
271	Aradu.CE2Z4	UDP-Glycosyltransferase superfamily protein
272	Aradu.CH6U0	LETM1-like protein
273	Aradu.CLQ9M	nitrate transporter 1.7
274	Aradu.CLY7T	late embryogenesis abundant protein
275	Aradu.CM87L	disease resistance protein (TIR-NBS-LRR class), putative
276	Aradu.CR9NG	receptor-like protein kinase 2
277	Aradu.CT0FI	receptor kinase 2
278	Aradu.CT448	myb transcription factor
279	Aradu.CT5EJ	Protein phosphatase 2C family protein
280	Aradu.CXW2P	cysteine-rich RLK (RECEPTOR-like protein kinase) 26
281	Aradu.CZL81	subtilisin-like serine protease 2
282	Aradu.D03W8	Protein kinase superfamily protein
283	Aradu.D0NVI	receptor-like serine
284	Aradu.D3M3F	L-ascorbate oxidase [Glycine max]
285	Aradu.DAC4M	PLATZ transcription factor family protein
286	Aradu.DAV01	Peroxidase superfamily protein
287	Aradu.DE5X6	Protein kinase superfamily protein
288	Aradu.DF39S	cinnamyl alcohol dehydrogenase 9
289	Aradu.DGR2N	DOF zinc finger protein 2
290	Aradu.DKR1D	SAUR-like auxin-responsive protein family
291	Aradu.DLG8U	heat shock protein 70
292	Aradu.DN10D	calmodulin-binding family protein
293	Aradu.DP2D5	basic helix-loop-helix (bHLH) DNA-binding superfamily protein
294	Aradu.DSS3T	Cell wall protein Exp1 n
295	Aradu.DT3LR	Protein kinase superfamily protein
296	Aradu.DW9XI	MYB transcription factor MYB48 [Glycine max]
297	Aradu.DZ5Y1	proline-rich protein 4
298	Aradu.E03PA	uncharacterized protein LOC100788162 isoform X3 [Glycine max]
299	Aradu.E0DJP	F-box
300	Aradu.E0ZB7	Disease resistance protein (TIR-NBS-LRR class) family
301	Aradu.E1MX8	Cell wall protein Exp4 n
302	Aradu.E252Y	receptor-like serine
303	Aradu.E2TII	ethylene-responsive transcription factor 4-like [Glycine max]
304	Aradu.E36HP	null
305	Aradu.E5U8P	Plant invertase
306	Aradu.E740H	heat shock transcription factor B3
307	Aradu.EE1XG	receptor-like protein kinase 4
308	Aradu.EF0RS	F-box
309	Aradu.EHP84	receptor-like serine
310	Aradu.EMG6I	Pathogenesis-related thaumatin superfamily protein
311	Aradu.EQ9K6	FAD-binding Berberine family protein
312	Aradu.ET296	beta-fructofuranosidase
313	Aradu.EU3KX	Sulfite exporter TauE
314	Aradu.EX55Q	Protein kinase family protein
315	Aradu.EXN4Y	heat shock protein 70
316	Aradu.EY4MD	WRKY family transcription factor
317	Aradu.EZ1CV	disease resistance protein (TIR-NBS-LRR class)
318	Aradu.EZ8L5	nitrate transporter 1.1

№	Gene model	Annotation
319	Aradu.EZY2W	Disease resistance protein (TIR-NBS-LRR class) family
320	Aradu.F06JI	ferrochelatase 1
321	Aradu.F2VIG	aldehyde dehydrogenase family 2 member C4-like [Glycine max]
322	Aradu.F7E8U	UDP-Glycosyltransferase superfamily protein
323	Aradu.F8RAG	myb transcription factor
324	Aradu.FA6IJ	Cytochrome P450 superfamily protein
325	Aradu.FB2F2	respiratory burst oxidase homolog B
326	Aradu.FBB2P	TRAM, LAG1 and CLN8 (TLC) lipid-sensing domain containing protein
327	Aradu.FCN11	disease resistance protein (TIR-NBS-LRR class), putative
328	Aradu.FDG48	MATE efflux family protein
329	Aradu.FJQ8M	60S ribosomal protein L27-1
330	Aradu.FM0MF	Cytochrome P450 superfamily protein
331	Aradu.FM6UE	myb transcription factor
332	Aradu.FXP12	4-hydroxyphenylpyruvate dioxygenase
333	Aradu.FXX49	Protein kinase superfamily protein
334	Aradu.FZ269	phenylalanine ammonia-lyase 2
335	Aradu.G2KK1	receptor-like serine
336	Aradu.G6ZA5	heat shock protein 70
337	Aradu.G78T6	disease resistance protein (TIR-NBS-LRR class), putative
338	Aradu.G7BKV	receptor-like protein kinase 2
339	Aradu.G7CZU	Cytochrome P450 superfamily protein
340	Aradu.G7UXI	myb transcription factor
341	Aradu.G85IC	blue copper protein-like [Glycine max]
342	Aradu.G87Z6	RING
343	Aradu.G9WGP	Disease resistance protein (TIR-NBS-LRR class) family
344	Aradu.GBG51	haloacid dehalogenase-like hydrolase
345	Aradu.GD2QE	protein serine
346	Aradu.GD6AN	Calmodulin binding protein-like
347	Aradu.GE19E	sucrose synthase 3
348	Aradu.GI8Z2	WRKY family transcription factor
349	Aradu.GJG28	Chitinase family protein
350	Aradu.GM1B4	peptide transporter 1
351	Aradu.GM66A	receptor-like protein kinase 2
352	Aradu.GP5CS	disease resistance protein (TIR-NBS-LRR class), putative
353	Aradu.GP6IB	cinnamyl alcohol dehydrogenase 9
354	Aradu.GXX4P	nudix hydrolase homolog 18
355	Aradu.H0G14	12-oxophytodienoate reductase 1
356	Aradu.H128Z	receptor-like protein kinase 2
357	Aradu.H2LY1	putative calcium-transporting ATPase 13, plasma membrane-type-like [Glycine max]
358	Aradu.H5DWF	LRR receptor-like kinase
359	Aradu.H5REB	heat shock transcription factor B4
360	Aradu.H96P1	Cytochrome P450 superfamily protein
361	Aradu.HB34E	lectin protein kinase family protein
362	Aradu.HJZ2Z	myb transcription factor
363	Aradu.HK5Y3	3-ketoacyl-CoA synthase 1
364	Aradu.HM4L0	disease resistance protein (TIR-NBS-LRR class), putative
365	Aradu.HN9N9	putative indole-3-acetic acid-amido synthetase GH3.9
366	Aradu.HP6LS	null
367	Aradu.HP9JD	Cytochrome P450 superfamily protein
368	Aradu.HS5Y0	acetyltransferase (GNAT) domain protein
369	Aradu.HU3I3	probable peptide
370	Aradu.HY1E7	MATE efflux family protein
371	Aradu.I0027	phytochelatin synthase 2
372	Aradu.I0X0M	Protein kinase superfamily protein

№	Gene model	Annotation
373	Aradu.I2HPG	receptor-like protein kinase 2
374	Aradu.I2JEZ	glutathione S-transferase 6
375	Aradu.I31PQ	Protein kinase superfamily protein
376	Aradu.I3UUC	receptor-like protein kinase 2
377	Aradu.I7XQM	disease resistance protein (TIR-NBS-LRR class), putative
378	Aradu.I957I	Cytochrome P450 superfamily protein
379	Aradu.I9JU3	receptor-like kinase 1
380	Aradu.I9TTZ	ethylene-responsive transcription factor 1B
381	Aradu.I9VXB	PPPDE putative thiol peptidase family protein
382	Aradu.IB6P2	GATA transcription factor 9
383	Aradu.IFC7W	phytosulfokine 5 precursor
384	Aradu.IIE2D	plasma membrane intrinsic protein 2
385	Aradu.IJE02	receptor-like serine
386	Aradu.IL3D8	gibberellin 20 oxidase 1-like [Glycine max]
387	Aradu.ILB9Z	1-aminocyclopropane-1-carboxylate oxidase
388	Aradu.IM707	Protein kinase family protein
389	Aradu.IQ81A	scarecrow-like transcription factor PAT1-like [Glycine max]
390	Aradu.IS5YT	Transketolase
391	Aradu.ISN1L	DOF zinc finger protein 1
392	Aradu.IU1HH	phenylalanine ammonia-lyase 2
393	Aradu.IV8SJ	expansin A1
394	Aradu.IW9Q8	receptor-like serine
395	Aradu.IX5G5	sulfotransferase 2A
396	Aradu.IY0H8	ERD (early-responsive to dehydration stress) family protein
397	Aradu.IZ0B7	receptor kinase 1
398	Aradu.IZA6Y	E3 ubiquitin-protein ligase RMA1H1-like isoform X2 [Glycine max]
399	Aradu.J13DP	subtilisin-like serine protease 2
400	Aradu.J1VYR	nitrate transporter 1.1
401	Aradu.J2BV7	protein kinase family protein
402	Aradu.J4QJ9	disease resistance protein (TIR-NBS-LRR class), putative
403	Aradu.J4TNX	receptor serine
404	Aradu.J6188	Cytochrome P450 superfamily protein
405	Aradu.J6P55	3-ketoacyl-CoA synthase 11
406	Aradu.J77I5	Pectate lyase family protein
407	Aradu.J7KPQ	respiratory burst oxidase protein F
408	Aradu.J8H2F	DOF zinc finger protein 1
409	Aradu.J90Q6	cationic amino acid transporter 5
410	Aradu.J9JP2	chlorophyll A
411	Aradu.J9V4P	glutamate decarboxylase
412	Aradu.JA0HW	Cytochrome P450 superfamily protein
413	Aradu.JA8X8	receptor-like protein kinase 2
414	Aradu.JB051	Chitinase family protein
415	Aradu.JEC4J	ethylene-responsive transcription factor RAP2-10 [Glycine max]
416	Aradu.JGT0L	MYB transcription factor MYB60 [Glycine max]
417	Aradu.JJ9Q9	receptor-like protein kinase 2
418	Aradu.JKX0H	vacuolar cation
419	Aradu.JL6EF	heat shock protein 90.1
420	Aradu.JM7KB	glycerol-3-phosphate acyltransferase 2
421	Aradu.JTK38	respiratory burst oxidase homologue D
422	Aradu.JXL5S	serine carboxypeptidase-like 2
423	Aradu.JZ5YG	P-loop containing nucleoside triphosphate hydrolases superfamily protein
424	Aradu.JZT16	Cell wall protein Exp4 n
425	Aradu.K0SK7	serine carboxypeptidase-like 18

№	Gene model	Annotation
426	Aradu.K13LX	unknown protein
427	Aradu.K23U3	ACT domain repeat 8
428	Aradu.K2DQ9	heat shock transcription factor B4
429	Aradu.K3P5U	ankyrin repeat-containing protein 2
430	Aradu.K3RPT	FAD-binding Berberine family protein
431	Aradu.K3ZSF	Cell wall protein Exp4 n
432	Aradu.K51ZU	Cytochrome P450 superfamily protein
433	Aradu.K6UHT	Cytochrome P450 superfamily protein
434	Aradu.K70HA	receptor-like protein kinase 4
435	Aradu.K8V1Y	MYB transcription factor MYB48 [Glycine max]
436	Aradu.KEE43	WRKY family transcription factor
437	Aradu.KF6LM	Flavin-binding monooxygenase family protein
438	Aradu.KG41H	WRKY family transcription factor
439	Aradu.KJ2TW	F-box
440	Aradu.KN04R	ADP,ATP carrier protein 1, chloroplastic-like [Glycine max]
441	Aradu.KN90T	subtilisin-like serine protease 2
442	Aradu.KNV7E	CYSTM1 family protein B-like isoform X3 [Glycine max]
443	Aradu.KQ6NP	Cytochrome P450 superfamily protein
444	Aradu.KQV51	Xyloglucan endotransglucosylase
445	Aradu.KW0UC	CYCLIN D1
446	Aradu.KY0UG	unknown protein
447	Aradu.KZZ3J	glutathione S-transferase 6
448	Aradu.L0584	aldo
449	Aradu.L0ADG	Disease resistance protein (TIR-NBS-LRR class) family
450	Aradu.L2QXE	protein YLS7-like [Glycine max]
451	Aradu.L44LL	receptor-like protein kinase 2
452	Aradu.L51MW	disease resistance protein (TIR-NBS-LRR class), putative
453	Aradu.L5GC6	cellulose synthase like G1
454	Aradu.L8W67	heat stress transcription factor A-6b-like [Glycine max]
455	Aradu.LA151	uncharacterized protein LOC100786645 [Glycine max]
456	Aradu.LBR87	Tetratricopeptide repeat (TPR)-like superfamily protein
457	Aradu.LCH2B	nodulin MtN21
458	Aradu.LD7BF	ethylene-responsive transcription factor 1A-like [Glycine max]
459	Aradu.LDF1T	receptor kinase 2
460	Aradu.LE6W1	Cytochrome P450 superfamily protein
461	Aradu.LF5V6	receptor-like serine
462	Aradu.LI3DS	3-oxo-delta(4,5)-steroid 5-beta-reductase-like protein
463	Aradu.LLW1X	receptor-like protein kinase 2
464	Aradu.LM21Q	Brassinosteroid signalling positive regulator (BZR1) family protein
465	Aradu.LM4BA	Cytochrome P450 superfamily protein
466	Aradu.LMA8E	heat shock protein 70
467	Aradu.LMZ0Z	MATE efflux family protein
468	Aradu.LN1BK	receptor like protein 6
469	Aradu.LNK8S	linoleate 13S-lipoxygenase 2-1, related protein
470	Aradu.LQK8F	E3 ubiquitin-protein ligase COP1-like [Glycine max]
471	Aradu.LW36F	Protein kinase superfamily protein
472	Aradu.LZ2RQ	purine permease 10
473	Aradu.LZ48I	L-ascorbate oxidase [Glycine max]
474	Aradu.M1GCR	WRKY family transcription factor
475	Aradu.M35Z5	heat shock protein 70
476	Aradu.M4EHD	Arabidopsis phospholipase-like protein (PEARLI 4) family
477	Aradu.M5WCG	TSPO(outer membrane tryptophan-rich sensory protein)-related
478	Aradu.M7BX0	sucrose transporter 4

Nº	Gene model	Annotation
479	Aradu.M999T	zinc finger protein CONSTANS-LIKE 2-like [Glycine max]
480	Aradu.M9H2P	fatty acyl-CoA reductase 3-like [Glycine max]
481	Aradu.M9J7Q	Protein kinase superfamily protein
482	Aradu.MA5U7	1-aminocyclopropane-1-carboxylate oxidase
483	Aradu.MA5WH	heat shock transcription factor A2
484	Aradu.ME45Z	embryo defective 1273 protein, putative
485	Aradu.MI2LX	cinnamyl alcohol dehydrogenase 9
486	Aradu.MI3AU	Cytochrome P450 superfamily protein
487	Aradu.MJ134	Plant invertase
488	Aradu.MR7KV	cinnamyl alcohol dehydrogenase 9
489	Aradu.MRQ6G	Cytochrome P450 superfamily protein
490	Aradu.MS98X	heat shock protein 70
491	Aradu.MSX28	Cytochrome P450 superfamily protein
492	Aradu.MTS9G	aldehyde dehydrogenase family 2 member C4-like [Glycine max]
493	Aradu.MU0PR	disease resistance protein (TIR-NBS-LRR class), putative
494	Aradu.MU7BZ	Protein kinase superfamily protein
495	Aradu.MVB2G	GRAM domain-containing protein
496	Aradu.MZ49K	Cytochrome P450 superfamily protein
497	Aradu.MZ5UW	P-loop containing nucleoside triphosphate hydrolases superfamily protein
498	Aradu.N0ICS	receptor-like protein kinase 2
499	Aradu.N1UIU	receptor-like serine
500	Aradu.N2PLN	Brassinosteroid signalling positive regulator (BZR1) family protein
501	Aradu.N3KUA	Disease resistance protein (TIR-NBS-LRR class) family
502	Aradu.N56TJ	probable pectinesterase
503	Aradu.N5R7X	Cytochrome P450 superfamily protein
504	Aradu.N6W6K	receptor-like protein kinase 2
505	Aradu.N7TW9	nitrate transporter 1.7
506	Aradu.N8TLT	Disease resistance protein (TIR-NBS-LRR class) family
507	Aradu.NBK01	protein yippee-like isoform X2 [Glycine max]
508	Aradu.NCD56	cofactor assembly of complex C
509	Aradu.NE81M	Protein kinase superfamily protein
510	Aradu.NG4LQ	Protein phosphatase 2C family protein
511	Aradu.NH44H	WRKY family transcription factor
512	Aradu.NL1YQ	disease resistance protein (TIR-NBS-LRR class), putative
513	Aradu.NNP8F	phenylalanine ammonia-lyase 2
514	Aradu.NSL0R	myb transcription factor
515	Aradu.NUY55	uncharacterized protein LOC100808320 isoform X2 [Glycine max]
516	Aradu.P1TMX	Pentatricopeptide repeat (PPR) superfamily protein
517	Aradu.P2C14	Protein phosphatase 2C family protein
518	Aradu.P2M19	nitrate transporter 1.7
519	Aradu.P33NE	indole-3-acetic acid inducible 31
520	Aradu.P5PIT	disease resistance protein (TIR-NBS-LRR class), putative
521	Aradu.P6DLH	Disease resistance protein (TIR-NBS-LRR class) family
522	Aradu.P6KKE	unknown protein
523	Aradu.P6ZPS	xyloglucan endotransglucosylase
524	Aradu.P709D	GDSL-like Lipase
525	Aradu.P72BL	receptor-like protein kinase 2
526	Aradu.PBR53	formin 8
527	Aradu.PC3XG	NAD-dependent epimerase
528	Aradu.PCZ19	Protein kinase superfamily protein
529	Aradu.PG6LA	glucomannan 4-beta-mannosyltransferase 2-like [Glycine max]
530	Aradu.PK283	WRKY family transcription factor
531	Aradu.PKA69	ERD (early-responsive to dehydration stress) family protein
532	Aradu.PQW7I	Cytochrome P450 superfamily protein

№	Gene model	Annotation
533	Aradu.PV4QE	Protein kinase superfamily protein
534	Aradu.PVH6K	glycerol-3-phosphate acyltransferase 1
535	Aradu.PXM5A	UDP-glycosyltransferase 74 F1
536	Aradu.PXP20	receptor-like protein kinase 2
537	Aradu.Q09VN	Protein kinase superfamily protein
538	Aradu.Q0IZH	transmembrane amino acid transporter family protein
539	Aradu.Q1D8Z	Cytochrome P450 superfamily protein
540	Aradu.Q1VWH	peptide transporter 5
541	Aradu.Q21Y2	Protein kinase superfamily protein
542	Aradu.Q29FA	trehalose phosphate synthase
543	Aradu.Q3CBB	Plasma membrane mannitol transporter n
544	Aradu.Q5HZK	Protein kinase superfamily protein
545	Aradu.Q8MCV	Cytochrome P450 superfamily protein
546	Aradu.Q92MC	homeobox associated leucine zipper protein
547	Aradu.Q9E1W	Cytochrome P450 superfamily protein
548	Aradu.QA44E	Zinc ion transmembrane transporter n
549	Aradu.QCH7F	heat shock protein 70
550	Aradu.QD5DS	LRR receptor-like kinase
551	Aradu.QD7TL	receptor lectin kinase
552	Aradu.QG7T6	MLO-like protein 5-like [Glycine max]
553	Aradu.QHL93	peptide transporter 2
554	Aradu.QL2WJ	heat shock protein 70
555	Aradu.QNX3N	UDP-Glycosyltransferase superfamily protein
556	Aradu.QU3D9	WRKY family transcription factor family protein
557	Aradu.QX6TK	zinc finger (C2H2 type, AN1-like) family protein
558	Aradu.QX8KD	Cytochrome P450 superfamily protein
559	Aradu.QYM4C	Uncharacterised protein family (UPF0497)
560	Aradu.R0TBT	transcription factor RADIALIS-like [Glycine max]
561	Aradu.R4V51	disease resistance protein (TIR-NBS-LRR class), putative
562	Aradu.R5UY0	MATE efflux family protein
563	Aradu.R6IE0	Water-selective transport intrinsic membrane protein 1 n
564	Aradu.R7CC6	DNA-binding protein n
565	Aradu.R8CQU	response regulator 3
566	Aradu.R8J0X	Protein kinase superfamily protein
567	Aradu.R8T0J	vacuolar protein sorting-associated protein 28 homolog 1
568	Aradu.R8UZU	receptor-like serine
569	Aradu.R99HZ	heat shock transcription factor A2
570	Aradu.R9SW4	2-oxoglutarate (2OG) and Fe(II)-dependent oxygenase superfamily protein
571	Aradu.RBU21	3-ketoacyl-CoA synthase 4
572	Aradu.RDK4X	probable pectinesterase
573	Aradu.RDQ06	Chitinase
574	Aradu.RER3E	heat shock protein 70
575	Aradu.RH9X1	peptide transporter 3
576	Aradu.RVF1V	heat shock transcription factor A3
577	Aradu.RY2KR	Glutamyl-tRNA reductase family protein
578	Aradu.RZ9Z0	WRKY family transcription factor
579	Aradu.S156I	zinc finger protein CONSTANS-LIKE 2-like [Glycine max]
580	Aradu.S2SYE	Cell wall protein EXP3 n
581	Aradu.S3B69	receptor-like protein kinase 2
582	Aradu.S3HBK	peptide transporter 1
583	Aradu.S4DGV	heat shock transcription factor B4
584	Aradu.S4DK6	receptor-like serine
585	Aradu.S6DQM	pectinesterase

№	Gene model	Annotation
586	Aradu.S7YD6	WRKY family transcription factor family protein
587	Aradu.SB5CI	probable calcium-binding protein CML41-like [Glycine max]
588	Aradu.SC6RY	GATA transcription factor 9
589	Aradu.SC9VF	Chaperone DnaJ-domain superfamily protein
590	Aradu.SG97P	Cytochrome P450 superfamily protein
591	Aradu.SJ3F5	F-box
592	Aradu.SL05Q	septum-promoting GTP-binding protein 1-like [Glycine max]
593	Aradu.SL2ND	guanine nucleotide-binding protein alpha-2 subunit isoform X3 [Glycine max]
594	Aradu.SM24D	MYB transcription factor MYB51 [Glycine max]
595	Aradu.SSI0J	glucan endo-1,3-beta-glucosidase-like [Glycine max]
596	Aradu.STQ8Y	uncharacterized protein LOC100800289 isoform X1 [Glycine max]
597	Aradu.T0GAI	Cytochrome P450 superfamily protein
598	Aradu.T2QXI	LETM1-like protein
599	Aradu.T2SMW	receptor-like protein kinase 2
600	Aradu.T37GN	calcium-transporting ATPase 2, plasma membrane-type protein
601	Aradu.T3V5H	receptor-like protein kinase 2
602	Aradu.T5517	Cytochrome P450 superfamily protein
603	Aradu.T5FHF	beta-fructofuranosidase
604	Aradu.T6VPT	Cell wall protein-like n
605	Aradu.T7TUD	purine permease 10
606	Aradu.T8C1S	Plasma membrane mannitol transporter n
607	Aradu.T955X	dehydration-responsive protein RD22
608	Aradu.TBP46	peptide transporter 1
609	Aradu.TFD03	blue copper protein-like [Glycine max]
610	Aradu.TH6UR	Cytochrome P450 superfamily protein
611	Aradu.TIH7T	uncharacterized protein LOC100780634 isoform X2 [Glycine max]
612	Aradu.TJ0ZU	Cytochrome P450 superfamily protein
613	Aradu.TL55R	protein kinase family protein
614	Aradu.TLI73	TWIN LOV protein
615	Aradu.TNI2G	Cytochrome P450 superfamily protein
616	Aradu.TP3KU	RING-H2 finger protein 2B
617	Aradu.TQE9X	receptor-like protein kinase 2
618	Aradu.TW1EN	Chitinase family protein
619	Aradu.TZ9RJ	Glutathione S-transferase family protein
620	Aradu.TZD57	Cytochrome P450 superfamily protein
621	Aradu.U21Z6	UDP-Glycosyltransferase superfamily protein
622	Aradu.U223I	null
623	Aradu.U3WE4	Calcium-binding EF-hand family protein
624	Aradu.U4BEA	UDP-glycosyltransferase 74 F1
625	Aradu.U6514	MYB transcription factor MYB48 [Glycine max]
626	Aradu.U6WKW	U-box domain-containing protein 45-like [Glycine max]
627	Aradu.U86RL	12-oxophytodienoate reductase 2
628	Aradu.U8TGW	Protein kinase superfamily protein
629	Aradu.UDE9J	probable pectinesterase
630	Aradu.UI0XD	cationic amino acid transporter 5
631	Aradu.UI1WY	Protein kinase superfamily protein
632	Aradu.UK6QI	Cytochrome P450 superfamily protein
633	Aradu.UL23E	gamma-glutamyl transpeptidase 4
634	Aradu.ULT0F	transmembrane amino acid transporter family protein
635	Aradu.UT1SE	FAD-binding Berberine family protein
636	Aradu.UW0DX	Protein kinase superfamily protein
637	Aradu.UX1RD	probable non-specific lipid-transfer protein 1-like [Glycine max]
638	Aradu.UX7L3	unknown protein
639	Aradu.UXN6T	NAC domain protein,
640	Aradu.UY6CA	receptor kinase 2

№	Gene model	Annotation
641	Aradu.UYV4P	FASCICLIN-like arabinogalactan-protein 11
642	Aradu.UZ2DQ	FAD-binding Berberine family protein
643	Aradu.V05DH	receptor-like protein kinase 2
644	Aradu.V2Z0U	Papain family cysteine protease
645	Aradu.V4MUL	null
646	Aradu.V5WI7	aldose 1-epimerase-like [Glycine max]
647	Aradu.V6VEV	receptor-like serine
648	Aradu.V7KPZ	Phosphate-responsive 1 family protein
649	Aradu.V8K6B	plasma membrane intrinsic protein 1C
650	Aradu.V9D7S	zinc finger protein CONSTANS-LIKE 5-like [Glycine max]
651	Aradu.V9VEN	putative phospholipid-transporting ATPase 9-like isoform X1 [Glycine max]
652	Aradu.VE705	WRKY family transcription factor
653	Aradu.VG8XJ	uncharacterized protein LOC100788403 [Glycine max]
654	Aradu.VHN28	probable pectinesterase
655	Aradu.VIF27	probable pectinesterase
656	Aradu.VK1JK	protein kinase 2A
657	Aradu.VK93Q	Protein kinase superfamily protein
658	Aradu.VL3IZ	WRKY family transcription factor
659	Aradu.VM730	Serine acetyl transferase n
660	Aradu.VMK7V	Sulfite exporter TauE
661	Aradu.VP7YH	receptor-like protein kinase 2
662	Aradu.VS60N	ethylene-responsive transcription factor 1B
663	Aradu.VSA8A	WRKY family transcription factor
664	Aradu.VSQ1I	uncharacterized protein LOC100804585 isoform X1 [Glycine max]
665	Aradu.VY606	receptor-like protein kinase 2
666	Aradu.W07KG	linoleate 13S-lipoxygenase 2-1, related protein
667	Aradu.W23YF	Adenine nucleotide alpha hydrolases-like superfamily protein
668	Aradu.W2C81	nitrate reductase 1
669	Aradu.W3B9W	disease resistance protein (TIR-NBS-LRR class), putative
670	Aradu.W3KTW	formin 8
671	Aradu.W51GD	basic 7S globulin [Glycine max]
672	Aradu.W64DR	beta-fructofuranosidase
673	Aradu.W67VC	Cytochrome P450 superfamily protein
674	Aradu.W7NWN	myo-inositol oxygenase 4
675	Aradu.WB5VJ	Lecithin:cholesterol acyltransferase family protein
676	Aradu.WBB7S	receptor lectin kinase
677	Aradu.WD394	receptor kinase 1
678	Aradu.WH49R	receptor-like protein kinase 2
679	Aradu.WH8HR	17.6 kDa class II heat shock protein
680	Aradu.WJ5JK	Cytochrome P450 superfamily protein
681	Aradu.WLE0A	Cell wall protein Exp4 n
682	Aradu.WM499	transcription factor RADIALIS-like [Glycine max]
683	Aradu.WWQ05	Cytochrome P450 superfamily protein
684	Aradu.X00D6	receptor-like serine
685	Aradu.X1BIM	Histone superfamily protein
686	Aradu.X3FXV	Cell wall protein Exp4 n
687	Aradu.X3GS1	ethylene-responsive transcription factor 12 [Glycine max]
688	Aradu.X4J41	Protein kinase superfamily protein
689	Aradu.X7ZD5	Cytochrome P450 superfamily protein
690	Aradu.X89BI	Actin cross-linking protein
691	Aradu.X91C4	glutamate decarboxylase 5
692	Aradu.X9N95	RING-H2 finger protein 2B
693	Aradu.XC1GR	GDSL-like Lipase
694	Aradu.XC65D	Disease resistance protein (TIR-NBS-LRR class) family
695	Aradu.XD25G	cysteine-rich receptor-like protein kinase 29-like [Glycine max]

Nº	Gene model	Annotation
696	Aradu.XF7S6	Plastid-lipid associated protein PAP
697	Aradu.XIP04	receptor-like protein kinase 2
698	Aradu.XM9I6	3-hydroxy-3-methylglutaryl-coenzyme A reductase-like protein
699	Aradu.XTS6M	receptor-like protein kinase 2
700	Aradu.XZB34	cotton fiber
701	Aradu.XZG8N	seed linoleate 9S-lipoxygenase
702	Aradu.Y1TID	xyloglucan endotransglucosylase
703	Aradu.Y4ACF	calmodulin-binding family protein
704	Aradu.Y5WI6	Protein kinase superfamily protein
705	Aradu.Y6LUX	Late embryogenesis abundant protein (LEA) family protein
706	Aradu.Y6X63	disease resistance protein (TIR-NBS-LRR class), putative
707	Aradu.Y7C8M	fatty acyl-CoA reductase 3-like [Glycine max]
708	Aradu.Y7IQR	protein YLS7-like [Glycine max]
709	Aradu.Y7TD6	Protein kinase superfamily protein
710	Aradu.Y7YB2	Cytochrome P450 superfamily protein
711	Aradu.Y9QIT	xyloglucan endotransglucosylase
712	Aradu.YA6WS	Cell wall protein Exp4 n
713	Aradu.YB9HI	ADP,ATP carrier protein 1, mitochondrial [Glycine max]
714	Aradu.YF1F6	RNA polymerase sigma factor
715	Aradu.YFQ3P	NAC domain containing protein 102
716	Aradu.YIQ80	NAC domain containing protein 19
717	Aradu.YK5P3	disease resistance protein (TIR-NBS-LRR class), putative
718	Aradu.YNN2E	GATA transcription factor 12
719	Aradu.YQI4L	GRAM domain-containing protein
720	Aradu.YU7JM	Disease resistance protein (TIR-NBS-LRR class) family
721	Aradu.YVT9V	Disease resistance protein (TIR-NBS-LRR class) family
722	Aradu.YXE38	Cytochrome P450 superfamily protein
723	Aradu.YY1QV	DOF zinc finger protein 1
724	Aradu.YZ9KD	receptor-like protein kinase 1
725	Aradu.Z0PKA	MATE efflux family protein
726	Aradu.Z2SQB	ferredoxin 3
727	Aradu.Z330P	response regulator 9
728	Aradu.Z3TSR	serine carboxypeptidase-like 7
729	Aradu.Z4BE5	receptor-like serine
730	Aradu.Z6I4Q	transcription factor bHLH63-like [Glycine max]
731	Aradu.Z86H5	CASP-like protein 7 [Glycine max]
732	Aradu.Z8E1G	UDP-Glycosyltransferase superfamily protein
733	Aradu.Z94KU	1-aminocyclopropane-1-carboxylate synthase 11
734	Aradu.Z9Z80	Glutamyl-tRNA reductase family protein
735	Aradu.ZD9KZ	Cell wall protein Exp4 n
736	Aradu.ZH02M	F-box
737	Aradu.ZHP56	glucan endo-1,3-beta-glucosidase-like [Glycine max]
738	Aradu.ZK8VV	galactinol synthase 1
739	Aradu.ZMM9X	xyloglucan endotransglucosylase
740	Aradu.ZPP4Z	Glutathione S-transferase family protein
741	Aradu.ZR4EL	Glutathione S-transferase family protein
742	Aradu.ZS0PF	LRR receptor-like kinase
743	Aradu.ZSF7K	Protein kinase superfamily protein
